# privateST: a feasible framework for privacy-preserving spatial transcriptomics prediction from histopathology images

**DOI:** 10.1038/s41598-026-55961-4

**Published:** 2026-06-03

**Authors:** Hakin Kim, Miran Kim, Buhm Han

**Affiliations:** 1 Interdisciplinary Program in Bioengineering, Seoul National University, Seoul, Republic of Korea; 2 Department of Mathematics, Hanyang University, Seoul, Republic of Korea; 3 Convergence Dementia Research Center, Seoul National University Medical Research Center, Seoul, Republic of Korea; 4 Department of Biomedical Sciences, BK21 Plus Biomedical Science Project, Seoul National University College of Medicine, Seoul, Republic of Korea

**Keywords:** Spatial transcriptomics, Homomorphic encryption, Resnet, Privacy-preservation, Histopathology, Deep learning, Computational biology and bioinformatics, Mathematics and computing

## Abstract

Predicting spatial transcriptomics from histology images offers cost-effective insights but faces privacy barriers preventing cross-institutional data sharing. To address this, we present privateST, a homomorphic-encryption-optimized framework that substantiates the feasibility of secure spatial transcriptomics prediction. To accommodate the computational constraints of homomorphic encryption, we downsampled the input images using bilinear interpolation. To improve prediction accuracy for the 100 target genes, we incorporated predictions for an auxiliary set of 150 highly expressed genes. This approach enables the model to learn from a more diverse genomic context, thereby refining the feature representations for the high-priority target genes. To adapt the architecture for homomorphic encryption, we replaced Max-Pooling and ReLU with Average-Pooling and polynomial approximation, respectively. These modifications eliminate non-linear comparison operations, significantly reducing the multiplicative depth. We also implemented multiplexed packing to optimize the efficiency of encrypted data processing. Remarkably, our results demonstrate that despite the reduced input resolution, this proposed approach achieves accuracy comparable to the original ResNet-18 configurations without downsampling.

**Supplementary Information:**

The online version contains supplementary material available at 10.1038/s41598-026-55961-4.

## Introduction

Recent advancements in deep learning have enabled the prediction of spatial transcriptomics (ST) data directly from standard Hematoxylin and Eosin (H&E) stained histopathology images^1–5^. This approach offers a substantial advantage by allowing clinical researchers to infer spatial gene expression without the need for costly experimental sequencing procedures. It enables researchers to leverage these inferred data in combination with other clinical and molecular datasets, thereby facilitating additional downstream analyses. Moreover, this approach also has the potential to improve the accuracy of patient prognosis.

However, the practical implementation of these models presents challenges for researchers, including the collection of large-scale training data and the challenges of applying such models in practice. A feasible alternative is to outsource the prediction task to a specialized cloud server equipped with a pre-trained prediction model. On this server, a user could upload a patient’s histopathology image to receive the corresponding predicted spatial transcriptomics data.

The primary barrier to adopting such a cloud-based workflow lies in concerns over patient privacy. Hospital data, in particular, is considered exceptionally sensitive, demanding a higher level of protection than general data^6^. This concern is also acute for histopathology images. Recent studies have revealed that histopathology images contain unique, patient-specific features that AI models can exploit to link images back to an individual, posing a significant privacy risk^7,8^. The handling of such sensitive patient information is governed by stringent legal frameworks. For example, the General Data Protection Regulation (GDPR) in Europe and the California Consumer Privacy Act (CCPA) in the United States impose strict requirements on data sharing^9–12^. Consequently, sharing raw patient data including histopathology images with external services is severely restricted.

To address the inherent privacy and technical barriers in spatial transcriptomics, we introduce privateST to demonstrate the feasibility of secure gene expression prediction in a zero-trust environment. Unlike standard deep learning approaches that necessitate access to unencrypted (plaintext) data, privateST performs inference directly on encrypted ciphertexts^13–15^. This capability is enabled by a homomorphic-encryption-optimized ResNet-18 backbone, specifically re-architected to overcome the arithmetic limitations of homomorphic encryption during complex neural inference. Key design strategies for our homomorphic-encryption-optimized architecture include:

1. Resolution Downsampling: To accommodate the computational constraints of homomorphic encryption, we downsampled the input images from $224 \times 224 \times 3$ to $64 \times 64 \times 3$ via bilinear interpolation. This reduction enables the entire input to be packed into a single ciphertext under standard homomorphic encryption (CKKS^16^) parameters ($N={2^{16}}$).
2. Linearization of Spatial Pooling: We replaced Max Pooling layers, which are nonlinear with Average Pooling to eliminate comparison operations that are computationally prohibitive in homomorphic encryption, relying instead on efficient arithmetic additions.
3. Polynomial Approximation: To approximate the non-linear ReLU function within the constraints of polynomial arithmetic, we utilized composite polynomials to maintain non-linear feature extraction capabilities.
4. Target Expansion Strategy: To mitigate the potential accuracy loss resulting from the aforementioned downsampling and architectural modifications, we adopted a strategy of predicting the top 250 highly expressed genes. By selecting the target 100 genes from this expanded set, we capture a more diverse genomic context, thereby achieving superior accuracy for the top 100 genes compared to the baseline model trained exclusively on the target subset. This approach utilizes the additional genes as auxiliary supervision to enrich the shared feature representation, thereby improving predictive fidelity for the target subset^17,18^.
5. Throughput Optimization: We employed a single-shot multiplexed packing strategy and the Baby-Step Giant-Step (BSGS) algorithm to maximize data density and minimize rotation overhead during inference.

We term this homomorphic-encryption-optimized model, which integrates the five design strategies outlined above, privateST. To further validate the robustness and generalizability of our approach, we extended our comparative analysis to include more complex architectures (EfficientNet-b0 and EfficientNet-b4). Our results demonstrate that privateST maintains competitive accuracy against these advanced baselines and more complex models. All cryptographic implementations and inference processes were developed using the Orion library^20^.

## Results

### Implementation of homomorphic-encryption-optimized ResNet

**Fig. 1:**
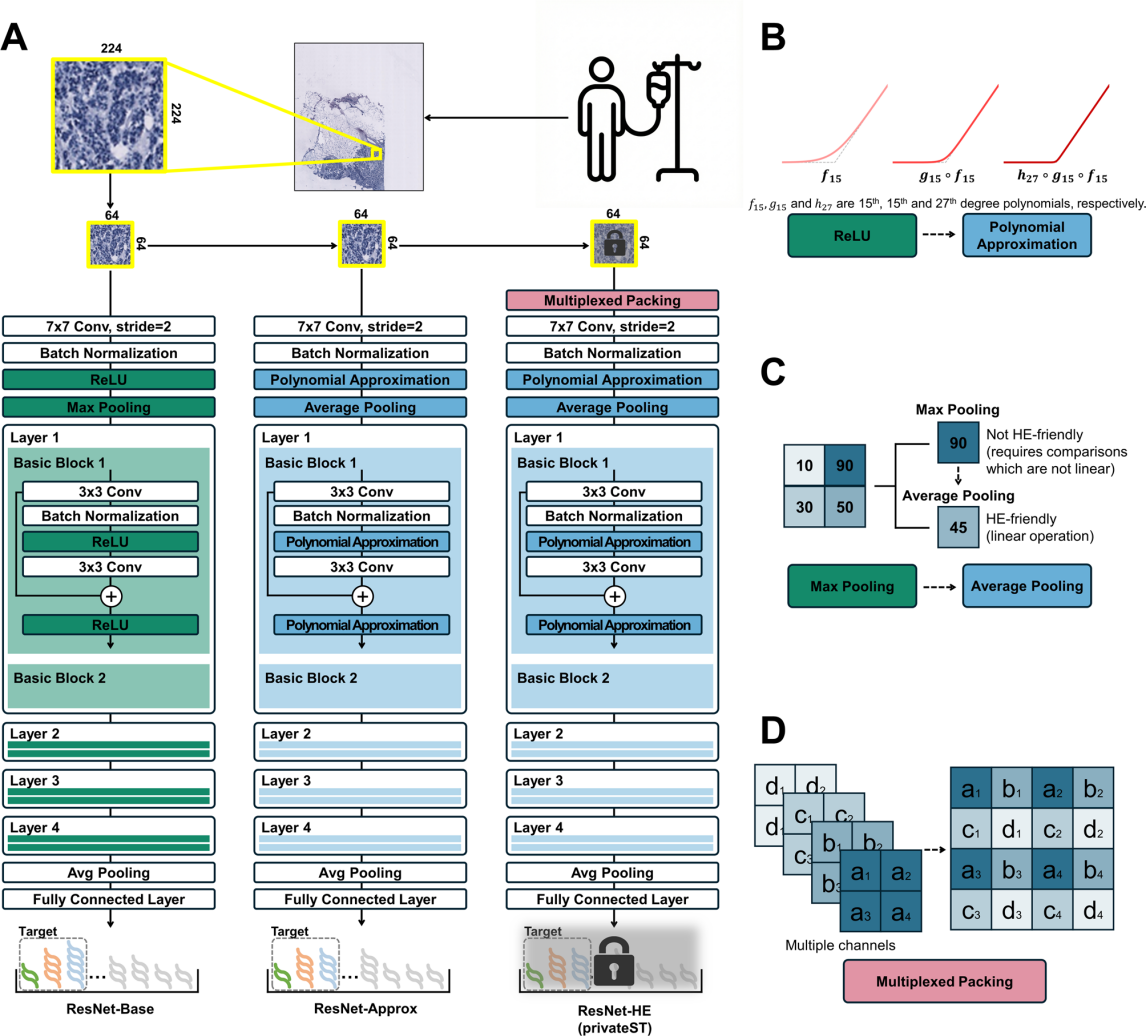
Overview of the privateST framework. (a) Comparison of architectures: standard Base, Approx (with homomorphic-encryption-friendly modifications), and HE (privateST). (b) Approximation of ReLU activation using polynomials to enable homomorphic computation. (c) Replacement of non-linear Max Pooling with linear Average Pooling. (d) Multiplexed packing strategy to encode multiple channels into a single ciphertext.

To implement privateST, we utilized the ResNet-18 architecture as a backbone for predicting gene expression from histological images (Fig. 1). Specifically, we cropped a $224 \times 224 \times 3$ sized patch corresponding to the target region of interest (ROI) from whole slide image and subsequently downsampled the patch to $64 \times 64 \times 3$. To ensure compatibility with homomorphic encryption operations, we modified the standard ResNet-18 architecture by replacing non-polynomial activation functions (ReLU) with polynomial approximations—specifically using a composition of 15th, 15th, and 27th-degree polynomials. This adaptation was necessary because the comparison with zero required by ReLU is not natively supported in homomorphic encryption. Additionally, we substituted Max Pooling with Average Pooling layers. This is because Max Pooling relies on comparison operations that are computationally expensive in the encrypted domain, whereas Average Pooling consists solely of efficient linear operations. Furthermore, we adopted a multiplexed packing strategy to maximize computational throughput during encrypted inference. To mitigate potential accuracy loss from input downsampling and architectural modification, we trained the model on an expanded set of the top 250 highly expressed genes. Utilizing the additional 150 genes as auxiliary supervision enriches the shared feature representation, thereby improving predictive fidelity for the primary 100 target genes.

To evaluate the performance and feasibility of our framework, we conducted a comparative analysis using three distinct model configurations (Fig. 1A).

1. Base: This model serves as the baseline, utilizing the standard ResNet-18 architecture with original non-linear operations, including ReLU activation and Max Pooling.
2. Approx: This configuration represents the homomorphic-encryption-friendly variant of the baseline evaluated on unencrypted data. To ensure compatibility with homomorphic encryption, we replaced the non-linear Max Pooling layers with linear Average Pooling and substituted ReLU functions with polynomial approximations (specifically using a composition of 15th, 15th, and 27th-degree polynomials).
3. HE: This is the fully deployed model operating in the encrypted domain. It applies actual homomorphic encryption to the Approx architecture and incorporates a multiplexed packing strategy to maximize computational throughput during inference.

Detailed model configurations and approximation methods are described in the “Methods” and Supplementary Note 3.

### Practicality of privateST

The clinical application of AI using histopathology images presents a significant privacy challenge. Indeed, re-identification is possible with substantial accuracy, even from anonymized histopathology images^7,8^. This risk is elevated when images originate from the same tissue block or resection. In such cases, the image itself can become a potential biometric identifier, enabling a linking attack that exposes sensitive health information.

**Fig. 2:**
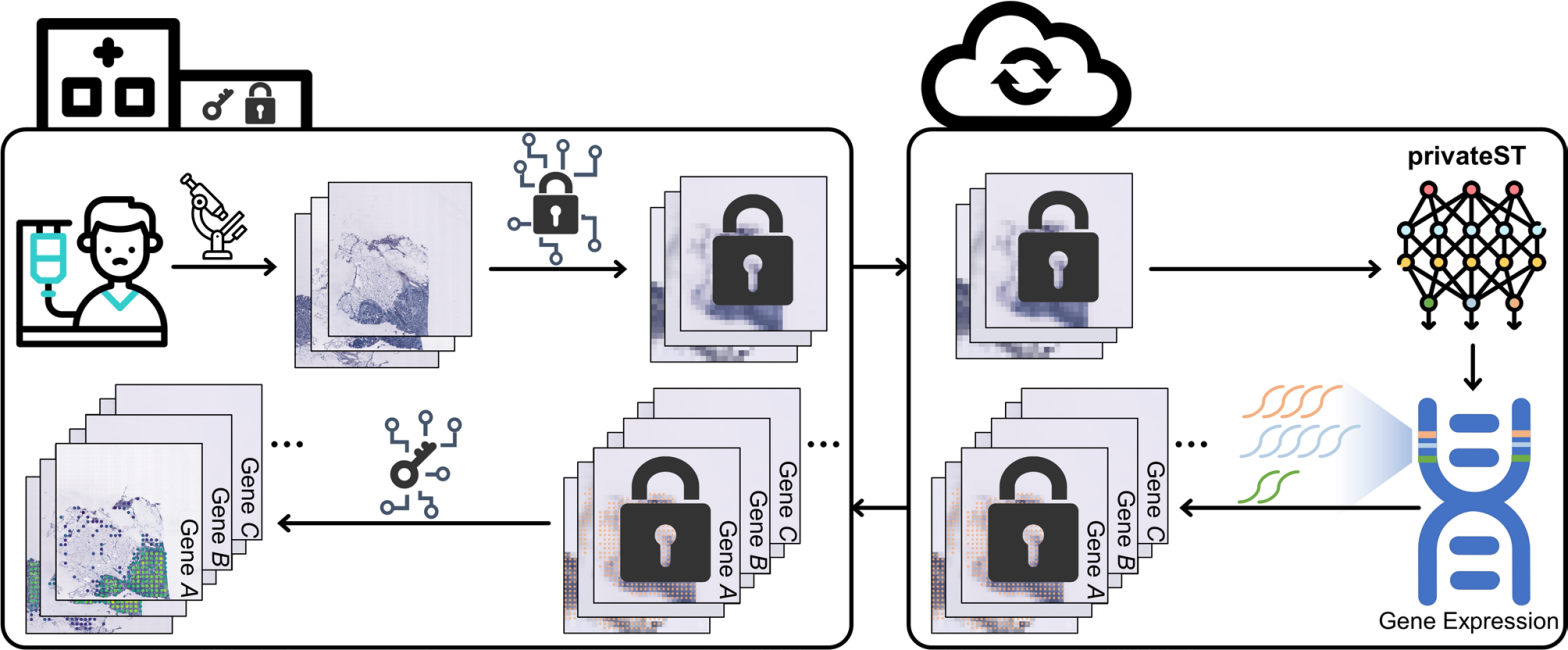
Workflow of how privates. The workflow begins with a hospital encrypting a Hematoxylin and Eosin image and transmitting the ciphertext to a server. The server performs inference directly on the encrypted data and returns an encrypted prediction. The hospital then decrypts the received data to obtain the predicted expression values for the target genes.

The privateST workflow, illustrated in Fig. 2, is designed to directly address this vulnerability and demonstrates a practical solution for secure inference. The process begins with the acquisition of sensitive histopathology images within a hospital. Specifically, the H&E images are tiled into patches corresponding to each spatial spot to prepare for the subsequent analysis. Each patch is then encrypted using public key and transmitted to an external server. On the server-side, privateST performs secure inference directly on the encrypted ciphertexts (encrypted patches), generating gene expression profiles without accessing the plaintexts data. The encrypted ciphertexts are then returned to the hospital and decrypted locally using the secret key. This workflow guarantees that the external server has access to neither the raw images nor the predicted data, thereby mitigating the re-identification risks demonstrated in recent literature. The execution of this entire workflow confirms the practicality of privateST as a practical tool for real-world clinical applications.

### Internal benchmarking and ablation study of ResNet-based architectures

To identify the optimal modeling strategy for predicting the top 100 target genes, we first benchmarked the three configurations (Base, Approx, and HE) established in Fig. 1. To this end, we conducted an analysis using the notation ${\mathrm{ResNet(}}{{\mathrm{S}}_{{\mathrm{crop}}}}{\mathrm{,}}{{\mathrm{S}}_{{\mathrm{in}}}}{\mathrm{)}}$. Here, ‘ResNet’ is used as a general notation encompassing all three variants-Base, Approx, and HE. ${{\mathrm{S}}_{{\mathrm{crop}}}}$ represents the patch crop size and ${{\mathrm{S}}_{{\mathrm{in}}}}$ represents the model input dimension obtained by resizing the cropped patch via bilinear interpolation. Notably, when ${{\mathrm{S}}_{{\mathrm{crop}}}}$ equals ${{\mathrm{S}}_{{\mathrm{in}}}}$, the original resolution is preserved without downsampling. Additionally, we trained the ResNet(224, 64) on an expanded set of 250 genes to enhance prediction for the top 100 targets^1^. We denote this configuration using a subscript for the gene count while omitting it for models trained on the standard 100 genes. For example, Base(64, 64) indicates a 64-sized crop used directly as input with standard ResNet-18 model without architectural modification, whereas ${\mathrm{Approx}}{\left( {{\mathrm{224,~64}}} \right)_{{\mathrm{250}}}}$ utilizes a 224-sized crop downsampled to 64 with architectural modification using the expanded 250-gene set. ${\mathrm{HE}}{\left( {{\mathrm{224,~64}}} \right)_{{\mathrm{250}}}}$ represents this modified architecture deployed on encrypted data using the expanded 250-gene set.

In this setup, the evaluation was conducted on a publicly available breast cancer spatial transcriptomics dataset^21^. We utilized 28,263 training spots from 22 patients and 529 test spots from a single patient (BC23287). Further details on the dataset are available in the “Dataset and preprocessing” section.

We adopted ${\mathrm{HE}}{\left( {{\mathrm{224,~64}}} \right)_{{\mathrm{250}}}}$ configuration for the final homomorphic encryption model. We designate this specific implementation as privateST. To identify the optimal modeling strategy for secure spatial transcriptomics, we conducted a comprehensive ablation study comparing privateST (${\mathrm{HE}}{\left( {{\mathrm{224,~64}}} \right)_{{\mathrm{250}}}}$) against 10 alternative configurations: (i) models without downsampling-Base(224,224) and Approx(224,224); (ii) downsampled models to dimension 64 with various input crop sizes- Base(512,64), Approx(512,64), Base(224,64), Approx(224,64), Base(64,64), and Approx(64,64); and (iii) models with an expanded 250-gene set- ${\mathrm{Base}}{\left( {{\mathrm{224,~64}}} \right)_{{\mathrm{250}}}}{\mathrm{~}}$and ${\mathrm{Approx}}{\left( {{\mathrm{224,~64}}} \right)_{{\mathrm{250}}}}$.

**Fig. 3:**
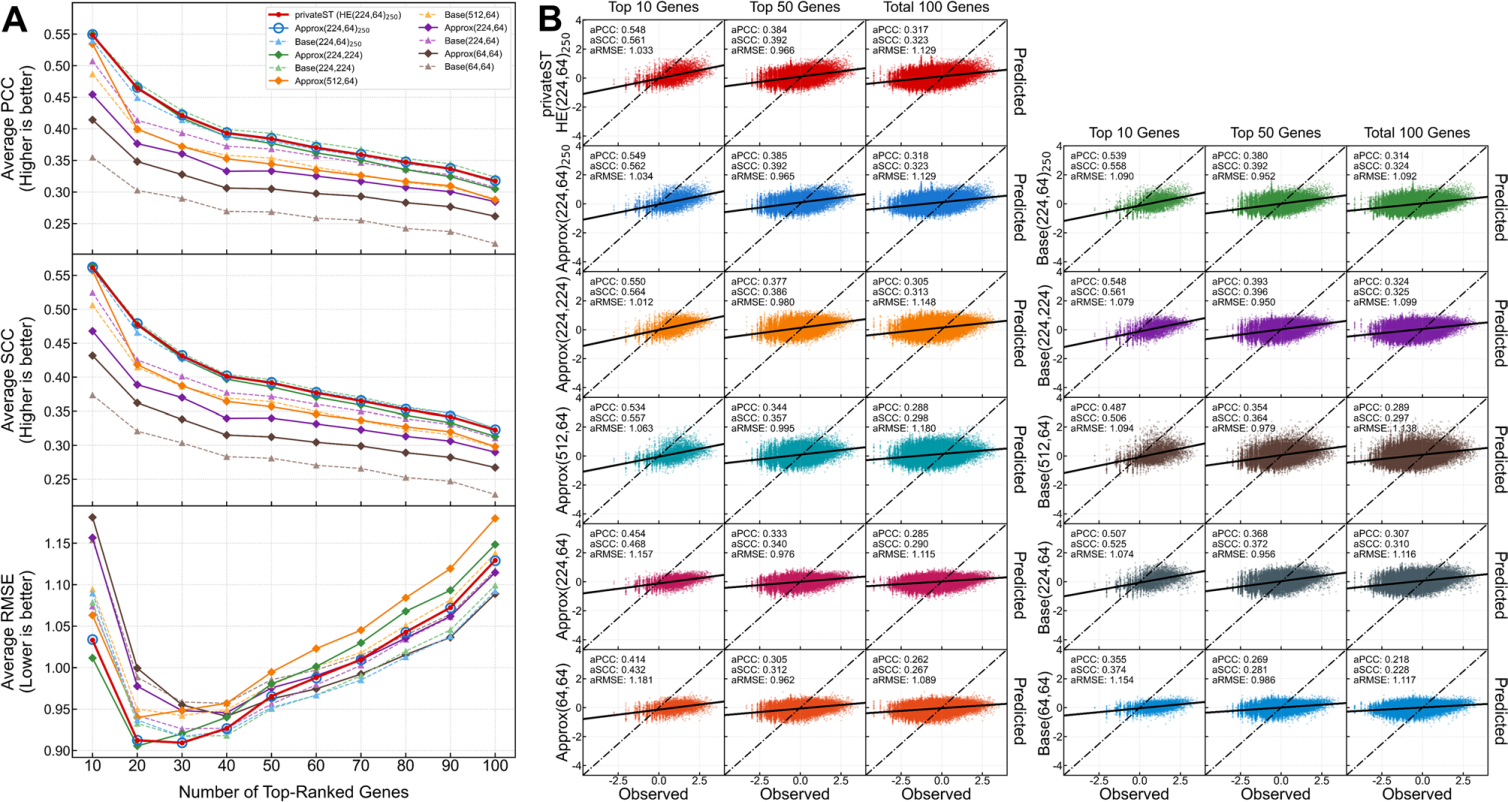
Internal benchmarking and ablation study of ResNet-based architectures. (a) Performance comparison across 11 model configurations as a function of the number of top-ranked genes. Three metrics are shown: Pearson Correlation Coefficient (PCC), Spearman Correlation Coefficient (SCC), and Root Mean Square Error (RMSE). privateST (red line) demonstrates competitive performance despite operating under homomorphic encryption constraints. The x-axis represents the number of top-ranked genes included in the evaluation, ranging from 10 to 100. (b) Scatter plots comparing observed versus predicted gene expression values for Top 10, Top 50, and Top 100 genes. The left panel displays Approx variants alongside privateST, while the right panel shows corresponding Base models. Dashed lines indicate the identity line (y = x), and solid lines represent regression fits. Each subplot displays average PCC, average SCC, and average RMSE values.

Figure 3A presents a comprehensive performance overview, illustrating metric trends, Pearson Correlation Coefficient (PCC), Spearman Correlation Coefficient (SCC), and Root Mean Square Error (RMSE). Note that the gene rankings in these plots are determined based on their mean normalized expression values in the test set (see “Methods” for details). Despite being specifically re-architected to accommodate the constraints of homomorphic encryption—notably by downsampling the input resolution and replacing non-linear operations (Max Pooling and ReLU)—privateST demonstrated competitive performance. privateST consistently achieved the highest PCC and SCC values among models downsampled to dimension 64. Regarding RMSE, while it remained within a competitive range across the total gene set, it demonstrated notably low error rates for the top-ranked genes, effectively capturing strong expression signals.

Our comparative analysis revealed four key insights regarding model behavior. First, we observed a negligible performance disparity between the unencrypted approximation (Approx) and the fully encrypted implementation (HE). Notably, ${\mathrm{HE}}{\left( {{\mathrm{224,~64}}} \right)_{{\mathrm{250}}}}$ and ${\mathrm{Approx}}{\left( {{\mathrm{224,~64}}} \right)_{{\mathrm{250}}}}$ achieved nearly identical PCC, SCC and RMSE values across various gene subsets, consistently exhibiting a minimal difference of only 0.001 at the third decimal place. This confirmed that, although the underlying CKKS scheme inherently introduces encryption and computation noise during homomorphic operations, its impact remains negligible in our evaluation. Second, regarding the choice of patch size under the fixed 64 input constraint, the intermediate patch size of 224 generally yielded better results than the broader 512 which undergoes heavy compression. This advantage was clearly observed in the baseline configurations, where Base(224,64) consistently outperformed Base(512,64) across all metrics. While the performance difference between Approx(224,64) and Approx(512,64) was negligible, ${\mathrm{Approx}}{\left( {{\mathrm{224,~64}}} \right)_{{\mathrm{250}}}}$ distinctly outperformed ${\mathrm{Approx}}{\left( {{\mathrm{512,~64}}} \right)_{{\mathrm{250}}}}$ (Supplementary Fig. S1). This suggests that when models are restricted to a reduced input dimension, optimal gene expression prediction requires a balanced field of view. Third, the performance gap resulting from replacing non-linear Max Pooling with linear Average Pooling (comparing Base and Approx) was not uniform across configurations: while Base(224,64) outperforms Approx(224,64), Base (64,64) underperforms its approximation counterpart. This indicates that sensitivity to linearization varies depending on the specific model architecture. Fourth, the Target Expansion Strategy proved highly effective: both privateST and ${\mathrm{Approx}}{\left( {{\mathrm{224,~64}}} \right)_{{\mathrm{250}}}},$trained on 250 genes, substantially outperformed Base(224,64) and Approx(224,64), trained on only 100 genes. This demonstrates that training on an expanded gene set captures richer genomic context, thereby improving prediction accuracy for the target genes.

In detail, Base(224,224) naturally serves as the high-fidelity upper bound for performance, as it operates on the native high-resolution input with standard non-linear operations (Max Pooling and ReLU), achieving an average PCC of 0.324 and an average RMSE of 1.099 for the target 100 genes. While the Base(224,224) sets the performance ceiling, privateST achieves competitive results with a PCC of 0.317 and an average RMSE of 1.129, effectively minimizing the accuracy gap despite the heavy cryptographic constraints. Critically, privateST consistently outperforms Approx(224,224) across all metrics, surpassing the approximate model which recorded an average PCC of 0.305 and an average RMSE of 1.148. This result is particularly significant given that privateST utilizes a strictly limited input size of 64. This restriction is dictated by the CKKS ring dimension $N={2^{16}}$. The native input size ($224 \times 224 \times 3$) is larger than ${2^{\left( {16 - 1} \right)}}$, making it impossible to encipher within a single ciphertext. Conversely, the reduced input size ($64 \times 64 \times 3$) is smaller than ${2^{16}}$, allowing the entire input to be efficiently packed into a single ciphertext. In contrast, Approx(224,224) benefits from the full native resolution of 224. This indicates that our Target Expansion Strategy—training on the top 250 genes to predict the top 100—effectively compensates for the information loss caused by reducing the input dimension. By capturing a broader genomic context, privateST recovers the accuracy lost during the linearization and downsampling processes, allowing it to surpass even the higher-resolution approximate model.

Figure 3B presents scatter plots comparing observed versus predicted gene expression values across all 11 model configurations, stratified by the number of top-ranked genes (Top 10, Top 50, and Total 100). The left panel displays the Approx variants alongside privateST, while the right panel shows the corresponding Base models.

The scatter distributions provide visual confirmation of the quantitative findings in Fig. 3A. The visual distribution of scatter points reveals distinct patterns related to crop size. Models with larger crop sizes (512) exhibit the widest scatter dispersion, followed by intermediate (224) and smallest (64) crop sizes. This progressive increase in point spread suggests that larger contextual fields introduce greater variance in predictions, though the intermediate crop size of 224 achieves the optimal balance between contextual information and prediction stability.

**Table 1:** Per-step PCC contribution of each privacy-driven modification, extracted from the ablation configurations.

Step	Modification	From	To	From aPCC	To aPCC	ΔaPCC
1	Downsampling	Base(224,224)	Base(224,64)	0.324	0.307	-0.017
2	Polynomial approximation and average pooling	Base(224,64)	Approx(224,64)	0.307	0.285	-0.022
3	Target Expansion Strategy	Approx(224,64)	${\mathrm{Approx}}{\left( {{\mathrm{224,~64}}} \right)_{{\mathrm{250}}}}$	0.285	0.318	+0.033
4	Encrypted inference	Approx${\left( {{\mathrm{224,~64}}} \right)_{{\mathrm{250}}}}$	${\mathrm{HE}}{\left( {{\mathrm{224,~64}}} \right)_{{\mathrm{250}}}}$	0.318	0.317	-0.001
	Cumulative net change	Base(224,224)	${\mathrm{HE}}{\left( {{\mathrm{224,~64}}} \right)_{{\mathrm{250}}}}$	0.324	0.317	-0.007

Values report the change in mean PCC over the top-100 target genes as each modification is added sequentially. The bottom row reports the cumulative net change from the plaintext Base(224,224) baseline to the fully encrypted ${\mathrm{HE}}{\left( {{\mathrm{224,~64}}} \right)_{{\mathrm{250}}}}$ configuration.

Across all models, the regression lines (solid) show slopes less than unity compared to the identity line (dashed, y = x), indicating a systematic tendency to underestimate extreme expression values. Lower-performing configurations such as Base(64,64) exhibit horizontally elongated scatter patterns, suggesting predictions concentrated near the mean with limited dynamic range.

Finally, as the number of target genes increases from Top 10 to Top 100, scatter points become progressively more dispersed across all models. This visual pattern reflects the decreasing predictability of lower-expressed genes, which likely exhibit weaker morphological associations in the histopathology images.

To make the attribution of performance loss explicit, we summarize the per-modification change in aPCC in Table 1, using the configuration labels shown in the figure. Starting from the plaintext Base(224,224) baseline (aPCC = 0.324), downsampling to Base(224,64) reduces aPCC to 0.307 (a decrease of 0.017), polynomial activation with average pooling at Approx(224,64) reduces aPCC to 0.285 (a decrease of 0.022), Target Expansion to 250 genes at ${\mathrm{Approx}}{\left( {{\mathrm{224,~64}}} \right)_{{\mathrm{250}}}}$ recovers aPCC to 0.318 (an increase of 0.033), and CKKS encrypted inference at ${\mathrm{HE}}{\left( {{\mathrm{224,~64}}} \right)_{{\mathrm{250}}}}$ yields aPCC = 0.317 (a decrease of 0.001). The cumulative net change relative to the plaintext baseline is a decrease of 0.007 in aPCC, demonstrating that the Target Expansion Strategy effectively absorbs the linearization cost.

### Effect of input downsampling resolution on predictive performance

To examine whether a higher input resolution would yield further accuracy gains beyond the $64 \times 64 \times 3$ setting adopted by privateST, we performed an additional ablation comparing the ${{64 \times 64 \times 3}}$ input against a $128 \times 128 \times 3$ input. We note that the $128 \times 128 \times 3$ setting cannot be directly deployed under the same CKKS parameters as privateST: a single ciphertext under our ring dimension $N={2^{16}}$ accommodates only ${2^{15}}=32,768$ plaintext slots, whereas a $128 \times 128 \times 3$ input requires *49,152~*slots and therefore cannot be packed into a single ciphertext. We therefore conducted this comparison at the plaintext Approx level. This is justified because the preceding ablation showed that the gap between Approx and HE is negligible (within 0.001 in aPCC), so the Approx-level comparison faithfully reflects what an encrypted $128 \times 128 \times 3$ deployment could at best achieve if the additional multi-ciphertext packing overhead were paid.

Under this setup, we trained two HE-compatible models sharing the same 224-pixel crop, the same 250-gene Target Expansion Strategy, and the same ResNet-18 backbone with polynomial activation and average pooling, differing only in the resize target applied via bilinear interpolation. Both configurations were evaluated on the same 529 test spots from patient BC23287. We denote these models as ${\mathrm{Approx}}{\left( {{\mathrm{224,~64}}} \right)_{{\mathrm{250}}}}$ and ${\mathrm{Approx}}{\left( {{\mathrm{224,~128}}} \right)_{{\mathrm{250}}}}$.

Performance metrics for both configurations are summarized in Supplementary Table S1. At the Top 10, Top 50, and Top 100 target genes, Approx ${\left( {{\mathrm{224,~64}}} \right)_{{\mathrm{250}}}}$ achieved aPCC of 0.549, 0.385, and 0.318, aSCC of 0.562, 0.392, and 0.323, and aRMSE of 1.034, 0.966, and 1.129. Under the same subsets, Approx ${\left( {{\mathrm{224,~128}}} \right)_{{\mathrm{250}}}}$ achieved aPCC of 0.533, 0.378, and 0.316, aSCC of 0.558, 0.393, and 0.328, and aRMSE of 1.059, 0.962, and 1.103. Overall, the two configurations exhibit comparable performance (absolute differences below 0.03 across all metrics and subsets), with the higher resolution yielding no consistent accuracy gain to justify its additional multi-ciphertext packing overhead under HE.

### Performance comparison with alternative model architectures

We compared privateST (${\mathrm{HE}}{\left( {{\mathrm{224,~64}}} \right)_{{\mathrm{250}}}}$) with non-ResNet architectures by evaluating quantitative performance and qualitative spatial expression patterns. Specifically, we benchmarked privateST against EfficientNet_b0 and EfficientNet_b4 architectures from the BrSTNet^1^ framework as comparison models, representing more complex models compared to our ResNet-18. We trained EfficientNet models by replicating the exact experimental setup described in the original paper^1^. This includes its auxiliary learning structure, a loss weight of 40, and the use of all remaining genes for the auxiliary task. All evaluations in this section were conducted using test patient BC23287. We note that this comparison is intended as a framework-level benchmark rather than a controlled comparison of backbone architectures. privateST differs from the EfficientNet baselines in several aspects—backbone, input resolution, HE-compatible modifications, and auxiliary learning structure—so in this comparison, the observed performance differences cannot be attributed to the backbone alone.

**Fig. 4:**
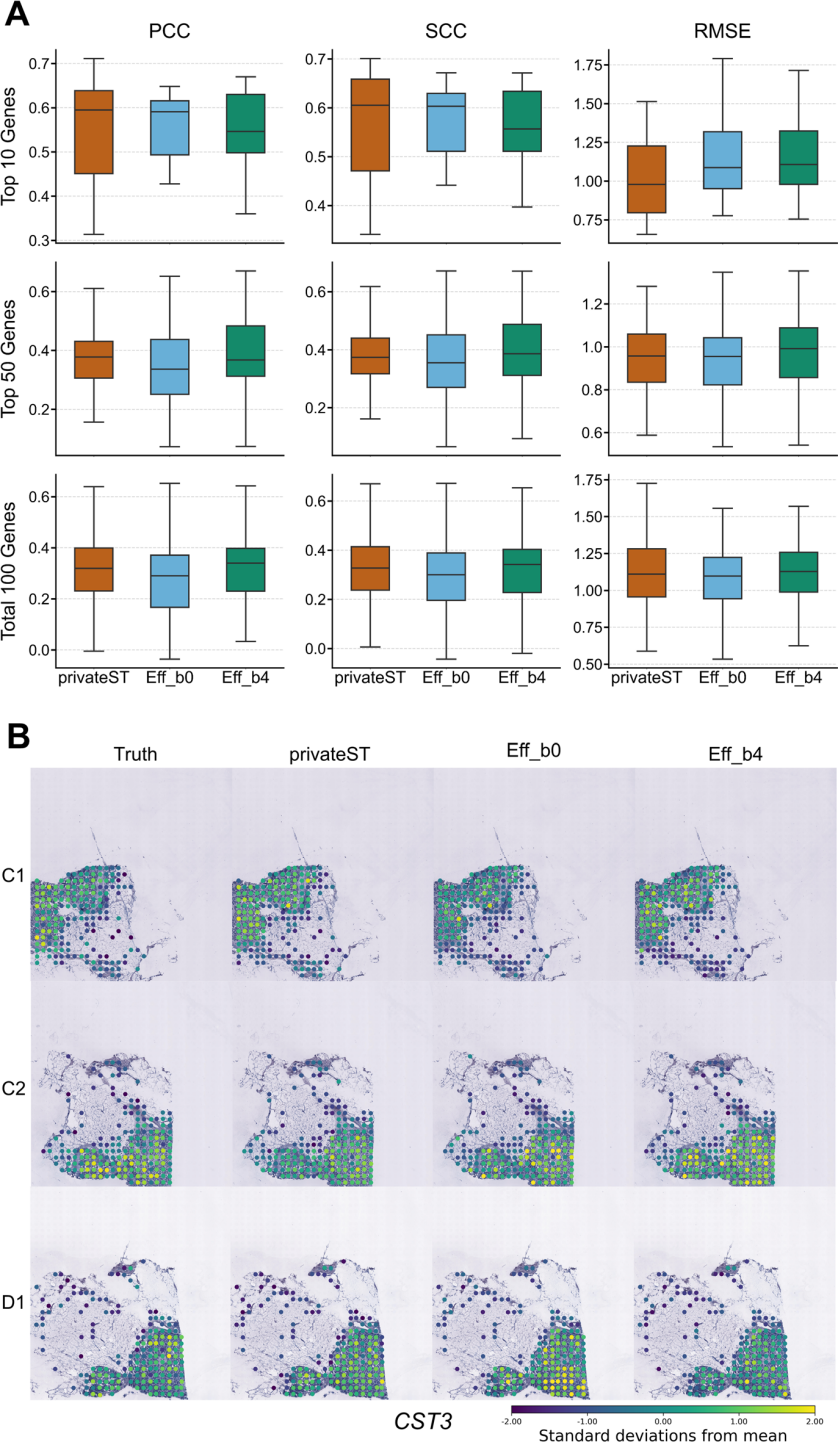
Performance comparison of privateST against alternative model architectures. (a) Quantitative evaluation of gene expression prediction performance across privateST, EfficientNet_b0 (Eff_b0), and EfficientNet_b4 (Eff_b4) models using test patient BC23287. Box plots display the distribution of Pearson Correlation Coefficient (PCC), Spearman Correlation Coefficient (SCC), and Root Mean Square Error (RMSE) for the top 10, top 50, and top 100 genes ranked by mean expression level. Each point represents an individual gene. (b) Spatial gene expression maps of *CST3* across three tissue sections (C1, C2, and D1) from the same test patient. Ground truth expression (“Truth”) is compared with predictions from privateST, EfficientNet_b0 (Eff_b0), and EfficientNet_b4 (Eff_b4). Expression values are shown as standard deviations from the mean.

First, we compared the quantitative metrics of privateST against EfficientNet models (Fig. 4A). The results were compared across three key metrics: Pearson Correlation Coefficient (PCC), Spearman Correlation Coefficient (SCC), and Root Mean Square Error (RMSE). Within each metric, we compared the performance across the top 10, 50, and 100 genes ranked by mean expression level.

Visual inspection of the box plots reveals a distinct trend in prediction variance. For the Top 10 genes, privateST exhibits a relatively wider interquartile range (IQR) for PCC and SCC, indicating higher variability in predictive performance for these high-signal targets. However, as the evaluation scope expands to the Top 100 genes, the variance for privateST markedly decreases, resulting in a tighter distribution compared to the EfficientNet models. This suggests that while privateST shows greater fluctuation in the highest-ranked genes, its predictive stability improves significantly across the broader gene set.

For the top 10 highly expressed genes, privateST achieved an average PCC of 0.549 and average SCC of 0.561, surpassing EfficientNet_b0 (aPCC: 0.526, aSCC: 0.544) while demonstrating performance comparable to EfficientNet_b4 (aPCC: 0.552, aSCC: 0.561). Notably, privateST demonstrated the lowest average RMSE (1.034) compared to EfficientNet_b0 (1.184) and EfficientNet_b4 (1.187), indicating superior prediction accuracy for highly expressed genes. For the full set of 100 genes, privateST maintained its advantage with an average PCC of 0.318 and an average SCC of 0.323. This performance surpassed EfficientNet_b0 (average PCC: 0.276, average SCC: 0.287) and remained competitive with EfficientNet_b4 (average PCC: 0.314, average SCC: 0.316). In terms of average RMSE, privateST (1.129) yielded a slightly higher error than EfficientNet_b0 (1.103) but successfully maintained a lower error compared to EfficientNet_b4 (1.144). The three models’ detailed metrics for all 100 genes are provided in Supplementary Table S2. For a more detailed gene-level analysis and PCC distribution, see Supplementary Note 1 and Supplementary Fig. S2. While the RMSE results for the full 100-gene set were comparable to the EfficientNet baselines, privateST consistently demonstrated superior capability in capturing spatial patterns. The consistently higher correlation metrics indicate that privateST effectively recovers expression trends and coherent tissue structures.

Second, we evaluated the qualitative spatial expression patterns of privateST with EfficientNet models (Fig. 4B). Specifically, we compared the spatial gene expression maps predicted by privateST and the comparison models (EfficientNet_b0 and EfficientNet_b4) with the ground truth. To facilitate a consistent comparison of spatial distributions across different models and genes, the predicted values were represented as standard deviations from the mean for each gene. As a representative example, we selected *CST3*, a gene encoding Cystatin C that has been widely implicated in breast cancer invasion and metastasis^22^. This gene showed consistently high predictive accuracy across all three models. Representative results for three tissue slides (C1, C2, and D1) from the test patient BC23287 are shown.

Across the tissue slides, the models exhibited distinct reconstruction characteristics relative to the ground truth. In tissue slides C1 and C2, privateST effectively captured the spatial coherence and intensity of high-expression zones, preserving the continuous tissue structure observed in the ground truth. In contrast, the EfficientNet models produced fragmented expression maps; while they correctly located the general regions, their predictions appeared discontinuous and scattered, particularly evident in the diffuse patterns of EfficientNet_b4 in slide C2. Similarly, in slide D1, privateST accurately identified the high-expression region as a cohesive domain, whereas EfficientNet models generated noisy, spotty distributions. These observations suggest a divergence in modeling characteristics: privateST excels at reconstructing continuous spatial domains consistent with tissue morphology, whereas EfficientNet models appear prone to local noise, resulting in less structurally coherent predictions.

### Performance degradation across the gene expression spectrum

**Fig. 5:**
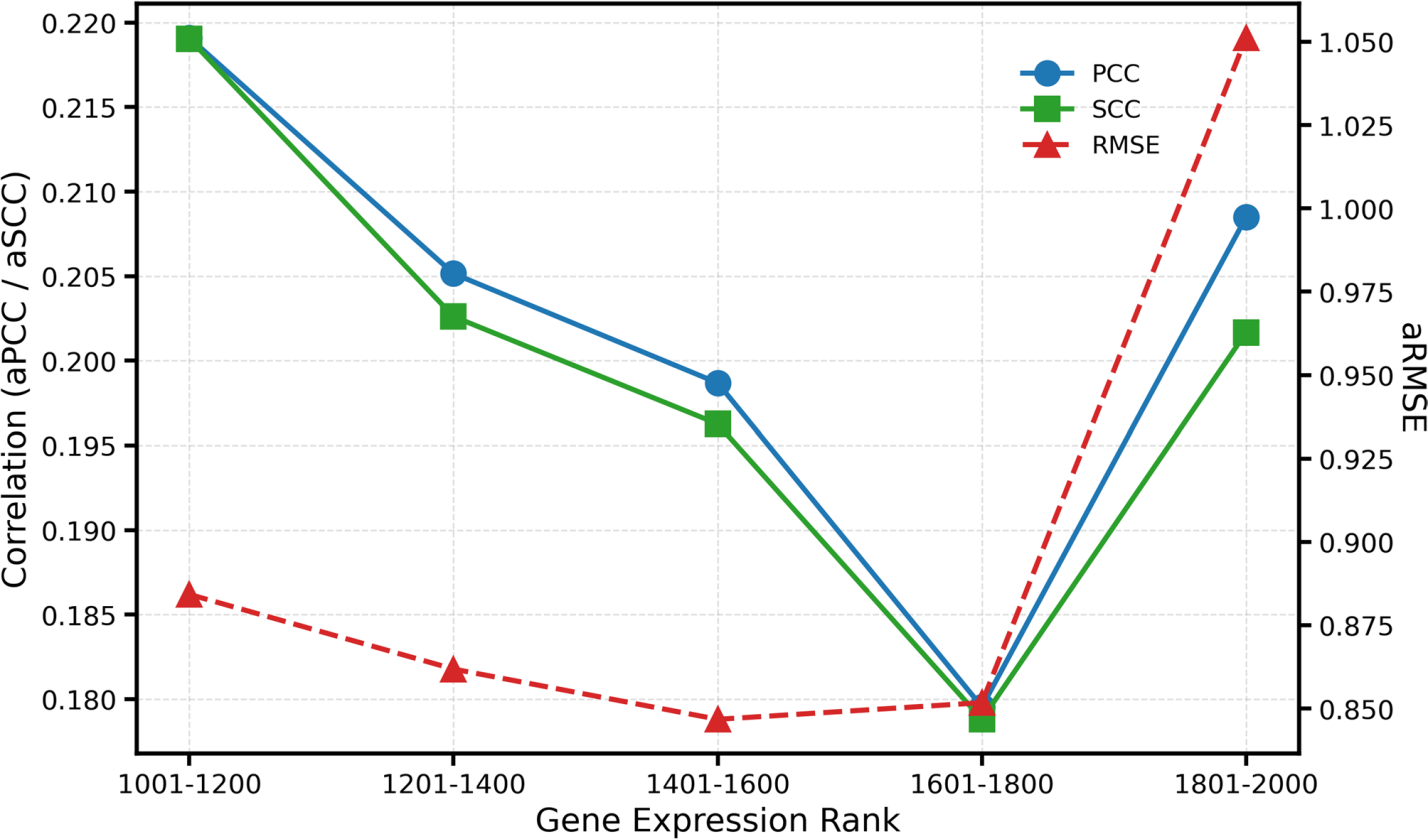
Performance degradation of privateST across the gene expression spectrum. Performance of privateST on test patient BC23287, extended to the top 2,000 highly expressed genes stratified into five bins of 400 genes (x-axis). Left y-axis: PCC (blue) and SCC (green). Right y-axis: RMSE (red, dashed). PCC and SCC decline monotonically with expression rank, while RMSE follows a non-monotonic trajectory due to z-score normalization interacting with zero-inflation in low-expression genes.

To characterize the model’s performance limits, we evaluated its predictive accuracy across five bins covering the gene ranks from 1,001 to 2,000 (Bins 1–5; Fig. 5). While aPCC and aSCC initially declined from 0.219 / 0.219 in Bin 1 (ranks 1,001–1,200) to 0.180 / 0.179 in Bin 4 (ranks 1,601–1,800), we observed a contradictory trend in the final bin. In Bin 5 (ranks 1,801–2,000), although the aPCC showed a slight rebound to 0.209, the aRMSE simultaneously surged to 1.051, the highest value recorded across all analyzed bins. This divergence between correlation and error metrics indicates a fundamental breakdown in predictive reliability. Despite a marginal increase in correlation, the high aRMSE demonstrates that the model fails to accurately estimate the absolute scale of gene expression in this range. The non-monotonic trajectory of aRMSE—which reached its lowest point of 0.847 in Bin 3 (ranks 1,401–1,600) before exploding in Bin 5—suggests that the model’s predictive capacity is highly unstable for low-expression genes. Consequently, the poor error metrics in the lowest bin confirm that morphology-based prediction becomes increasingly inaccurate as gene expression nears the noise floor. These results justify our primary focus on the top 100 genes, where the model maintains a more balanced and reliable performance across both correlation and error metrics.

### External validation on a different cancer type and tissue

**Fig. 6:**
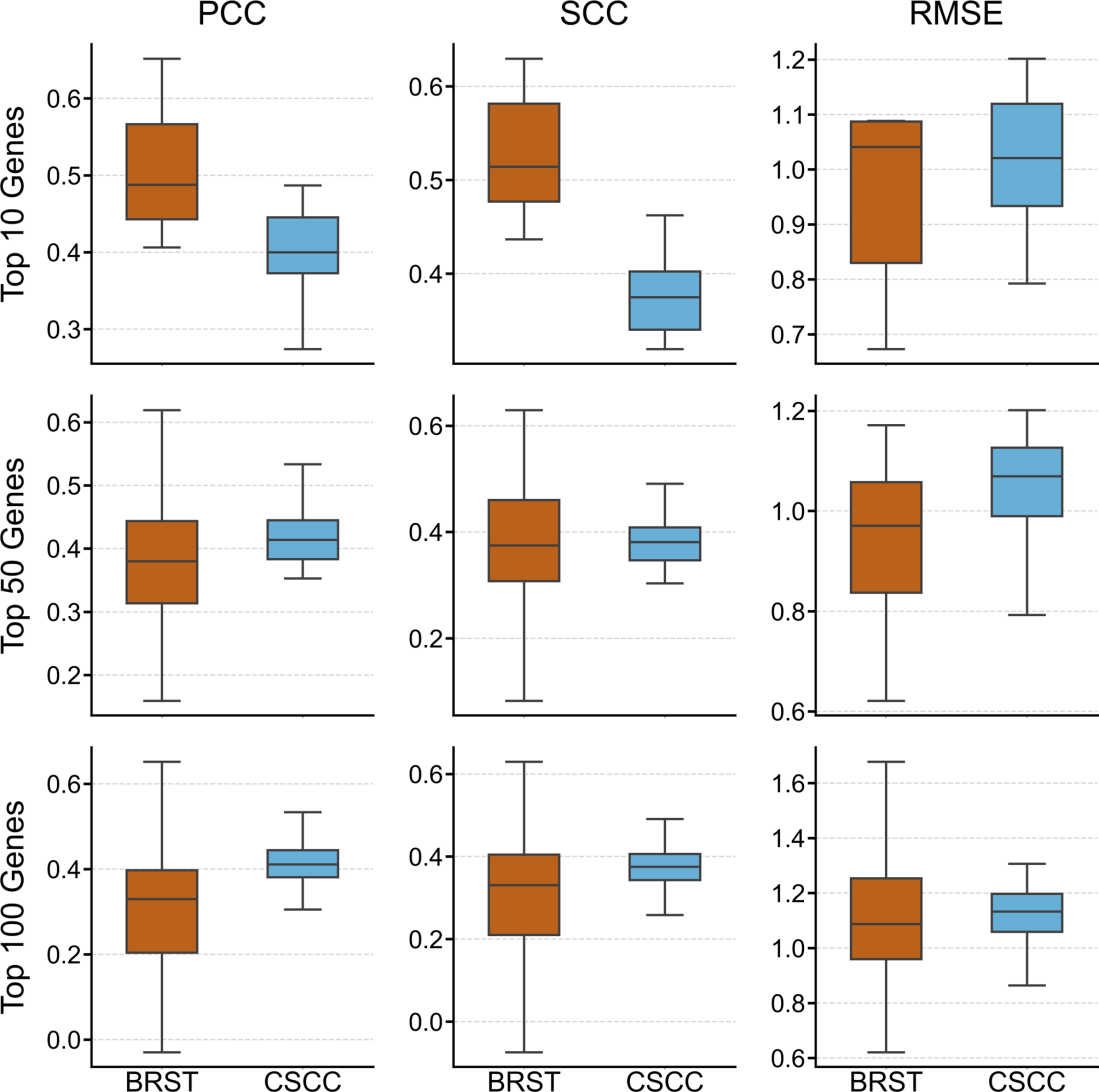
External validation of privateST on the CSCC dataset. Box plots show per-gene Pearson correlation coefficient (PCC), Spearman correlation coefficient (SCC), and root mean square error (RMSE) on the top 10, top 50, and top 100 most highly expressed genes for the BRST (breast cancer, orange) and CSCC (cutaneous squamous cell carcinoma, blue) datasets. The identical privateST pipeline, including the Target Expansion Strategy (top 100 + 150 auxiliary = 250 genes), CKKS-based encrypted inference, and polynomial-approximated activations, was applied to both datasets. privateST sustains prediction quality across both datasets, with slightly more stable performance on CSCC for the top 50 and top 100 gene subsets.

To assess generalizability beyond breast cancer, we further evaluated privateST on the cutaneous squamous cell carcinoma (CSCC) spatial transcriptomics dataset of Ji et al.^23^. CSCC arises in skin tissue — keratinocyte-based epidermis with dermal stroma — which is biologically distinct from the ductal/lobular glandular epithelium and adipose-rich stroma of breast tissue (BRST) evaluated above. We applied the identical privateST pipeline — preprocessing, Target Expansion Strategy (target 100 + 150 auxiliary = 250 genes), downsampling from $224 \times 224 \times 3$ to $64 \times 64 \times 3$ via bilinear interpolation, polynomial-approximated activation, and CKKS inference — without any architectural or hyperparameter retuning. As shown in Fig. 6, privateST sustained meaningful prediction quality on CSCC across all three metrics (PCC, SCC, RMSE) and all three gene subsets (top 10, top 50, top 100). Mean PCC and SCC values on CSCC were 0.40 / 0.37, 0.41 / 0.38, and 0.41 / 0.38 for the top 10, top 50, and top 100 genes, respectively. CSCC values were lower than the corresponding BRST results for the top 10 subset (BRST: 0.51 / 0.52), but matched or slightly exceeded BRST for the top 50 (BRST: 0.37 / 0.37) and top 100 (BRST: 0.31 / 0.31) subsets. The CSCC distributions also exhibited reduced inter-gene variance for the top 50 and top 100 subsets. These results indicate that the privateST pipeline generalizes across cancer types and biologically distinct tissues with the same architecture, although the absolute accuracy on the very-highest-expression genes can vary with the underlying tissue biology.

### Computational performance and resource overhead

To evaluate the computational overhead of homomorphic encryption in a practical setting, we benchmarked the performance of the privateST (${\mathrm{HE}}{\left( {{\mathrm{224,~64}}} \right)_{{\mathrm{250}}}}$), using the Seoul National University (SNU) Bio-AI SLURM Cluster. The evaluation was conducted on a node equipped with an Intel Xeon Platinum 8480 + processor at 2.00 GHz CPU, with the process configured to use 16 cores, and 512 GB of RAM. The software stack consisted of Python 3.13.2, PyTorch 2.6.0, and the Orion library^20^. The version of Orion used in this study was based on the state of its public GitHub repository as of September 16, 2025. Additionally, the system provides 128-bit security.

Under this environment, the prediction for a single encrypted image spot of size $64 \times 64 \times 3$ pixels took an average of 730.3 s (approximately 12.17 min), excluding encryption and decryption times. We measured the peak memory usage during the inference process and observed a maximum consumption of approximately 432 GB. Consequently, we recommend a system with at least 512 GB of RAM for stable operation.

This performance highlights a significant computational cost, which is the quantifiable privacy tax—the direct cost of ensuring the mathematical confidentiality of patient data. While this processing time is substantial, it demonstrates the current feasibility of the secure workflow and underscores the fundamental trade-off between data privacy and computational efficiency.

### Cryptographic configuration and resource scalability analysis

The adapted privateST model was compiled with carefully selected CKKS parameters to balance security, precision, and computational depth. We set the ring dimension (LogN) to 16, which defines a large ciphertext space ($N={2^{16}}$) to ensure both a high security level and sufficient data capacity for batch processing, as well as support for bootstrapping. To support the model’s deep architecture, we defined a ciphertext moduli chain (LogQ) of [55, 40, 40, 40, 40, 40, 40, 40, 40, 40, 40], providing the necessary computational budget for the required multiplicative depth. Furthermore, a scaling factor (LogScale) of 40 was employed to maintain high numerical precision for the gene expression values by scaling them by ${2^{40}}$ before encryption. Finally, a sparse secret key with a Hamming weight (H) of 192 was used to enhance the performance of encryption and decryption operations.

To further assess the impact of computational resources on performance, we conducted additional benchmarks on the SNU Bio-AI SLURM Cluster, varying system memory. To evaluate the effect of system memory, we tested the inference time using 112 CPU cores across several memory allocations: 512 GB, 1 TB, and 2 TB (providing 2,000,000 MB of memory after excluding system usage for 2 TB). The processing time remained stable with execution times of approximately 748.5, 723.4, and 749.6 s, respectively. This indicates that performance is not constrained by memory once the minimum requirement of approximately 432 GB is met.

### Analysis of data expansion and storage constraints

Homomorphic encryption, utilizing CKKS with a ring degree of $N={2^{16}}$ and a multiplicative depth of ${\mathrm{~}}L=10$, introduces a substantial data expansion of approximately 235 times, inflating a 48 KB ($64 \times 64 \times 3$) image to an 11.0 MB ciphertext. Given that our study utilizes 529 patches, retaining the original $224 \times 224 \times 3$ resolution (which is approximately 588 KB) would have resulted in a ciphertext size exceeding 134 MB per patch. This would impose a prohibitive burden on storage and bandwidth, rendering the workflow impractical. Therefore, our downsampling strategy achieves a 12x reduction in ciphertext size and plays a critical role in mitigating this privacy tax, thereby striking a necessary balance between prediction accuracy and the computational feasibility required for clinical deployment.

## Discussion

In this study, we introduced privateST to demonstrate the feasibility of using homomorphic encryption for the secure prediction of spatial transcriptomics, specifically addressing the critical vulnerability of patient re-identification in medical imaging. Secure inference on spatial transcriptomics had not previously been attempted, and privateST therefore provides an initial reference implementation against which subsequent methods can be benchmarked. This work extends approaches from the field of privacy-preserving genomic analysis, where methods have been developed for securely outsourcing tasks like Human Leukocyte Antigen (HLA) and Single Nucleotide Polymorphism (SNP) imputation^9,24,25^. While our results confirmed that secure inference is achievable, the direct application of standard deep learning models to the encrypted domain presents unique computational and arithmetic challenges.

First, to compensate for information loss from these approximations, we adopted a Target Expansion Strategy; training on a broader set of 250 genes enriched the feature representation, thereby recovering accuracy for the top 100 target genes. Second, we downsampled input images to $64 \times 64 \times 3$, enabling the entire input to be packed into a single ciphertext for reduced latency. Third, we replaced Max Pooling with Average Pooling to eliminate computationally prohibitive comparison operations, ensuring arithmetic compatibility. Fourth, we utilized composite polynomials to approximate ReLU, maintaining non-linear feature extraction capabilities within cryptographic constraints. Finally, we maximized throughput by employing single-shot multiplexed packing and the BSGS algorithm to minimize rotation overhead.

Beyond these architectural strategies, ensuring such high-level confidentiality inevitably incurs computational costs. The current per-spot runtime of 12.17 min scales to approximately 4.5 days per whole-slide image, sufficient to demonstrate feasibility but not acceptable for real-time clinical diagnostics. Hospital-scale deployment will therefore require a combination of (i) ROI-based selective inference, in which only diagnostically relevant patches are encrypted and transmitted, reducing encryption latency, bandwidth, and storage costs by an order of magnitude; and (ii) GPU-based CKKS acceleration, which has been shown to deliver approximately 100-fold speedups^26^, bringing per-slide runtime to roughly one hour. Accordingly, we position privateST as a feasibility demonstration rather than a deployment-ready clinical tool.

Looking ahead, the conceptual framework of privateST is not confined to predicting spatial transcriptomics from breast cancer histopathology. The core principle of performing inference on encrypted images is highly generalizable. For instance, beyond gene expression, the model could be trained to predict the presence or absence of cancer directly from histopathology patches, which is a less complex task. The framework of privateST could also be extended to a broader range of medical imaging modalities, such as Magnetic Resonance Imaging (MRI) and Computed Tomography (CT) scans. Such extensions would significantly broaden the impact of privacy-preserving machine learning in clinical settings, enabling secure multi-modal data analysis without compromising patient confidentiality.

Furthermore, the modular nature of our proposed approach allows for adaptability to rapid advancements in spatial transcriptomics. While current implementations rely on CNN-based backbones for computational efficiency within the encrypted domain, recent breakthroughs such as THOR^27^ demonstrate that homomorphic encryption is evolving to support complex Transformer-based operations. As these cryptographic optimizations mature, it may eventually become feasible to apply next-generation, single-cell resolution backbones^28^ within privacy-preserving frameworks, expanding the scope of secure genomic analysis. Beyond cryptographic advances, parallel progress in attention-based and language-model-driven representation learning has continued to reshape diverse application domains, ranging from weakly supervised semantic segmentation^29,30^ to large-language-model- and knowledge-graph-based recommendation^31,32^. As HE primitives mature to support more complex architectures, design principles emerging from these areas may inform future extensions of privateST toward richer, multi-modal models under encryption.

From a clinical perspective, the predicted spatial gene expression outputs of privateST carry direct relevance to multiple stages of patient care. The top-ranked target genes—such as *CST3*, which has been implicated in breast cancer invasion and metastasis^22^—provide spatially resolved molecular context that complements the morphological information available to pathologists. Such transcriptomic context can support diagnostic assessment by revealing regions enriched for tumor-associated expression patterns, prognostic evaluation by quantifying intra-tumor heterogeneity, and treatment selection by spatially localizing therapeutically targetable molecular features. Critically, privateST generates these outputs directly from routinely acquired H&E images, with all inference performed on encrypted data to preserve patient confidentiality. Conventional spatial transcriptomics, in contrast, requires tissue sectioning, library preparation, sequencing, and bioinformatic processing—steps whose cost and turnaround time currently limit clinical adoption. Together, these properties position privateST as a privacy-preserving framework that bridges morphological assessment and molecular profiling without exposing identifiable patient data and without additional experimental burden.

Beyond homomorphic encryption, the privacy-preserving machine learning landscape includes federated learning (FL), differential privacy (DP), and trusted execution environments (TEEs). FL and DP primarily protect training data—FL by avoiding raw-data sharing during collaborative training^33^, DP by injecting calibrated noise—but neither protects a patient sample submitted to a deployed model for prediction^34,35^. TEEs protect such samples at the prediction stage through hardware isolation but rely on vendor trust and remain vulnerable to side-channel attacks^36^. In contrast, privateST cryptographically protects the submitted patient sample and all intermediate computations during prediction, and requires no hardware trust. We therefore view privateST as complementary to FL and DP rather than competitive: FL and DP can be applied during training to leverage cross-institutional data and improve model accuracy^37^, while privateST serves the resulting model on encrypted patient samples at deployment, together forming an end-to-end privacy-preserving pipeline. Among methods that protect prediction-stage data, privateST and TEEs occupy distinct trade-offs: TEEs offer faster inference through hardware isolation but require vendor trust, whereas privateST trades computational overhead for cryptographic, zero-trust guarantees. As AI-driven diagnostics and large-scale medical imaging repositories become central to modern healthcare, the need for patient data security is paramount^38^. This requirement is especially acute for emerging precision medicine workflows — telemedicine and remote pathology consultation, cloud-based AI diagnostic services, and multi-institutional collaboration aimed at building diverse imaging datasets — where raw data sharing is often legally or ethically infeasible^35^. This concern intensifies in the emerging multi-omics era: as prediction increasingly integrates genomic, transcriptomic, and imaging data on cloud-based analytics platforms, each additional modality compounds the re-identification surface, and outsourced computation on aggregated patient data becomes the practical operational mode. In these settings, encrypted inference is a prerequisite for deployment rather than a privacy convenience. By integrating the principles of homomorphic encryption as demonstrated by privateST, such workflows can proceed without requiring hospitals to ever share raw patient data, advancing the promise of precision medicine on a global scale.

This study has several limitations. First, even with the GPU-acceleration roadmap discussed above, current per-spot latency remains incompatible with intraoperative or real-time diagnostic use cases, restricting privateST’s applicability to non-time-critical analyses. Second, the reported accuracy metrics are based on a filtered dataset focusing on spots with over 1,000 read counts and the top 100 most highly expressed genes, and performance may decrease for low-signal transcripts. Third, this study focused specifically on spatial gene expression prediction and has not been validated for other clinical predictive tasks such as multiclass classification^19^. Future research is required to determine the generalizability across a broader range of diagnostic applications. Fourth, evaluating our framework on a single test patient from the same breast cancer dataset limits the assessment of inter-patient variability and raises the risk of overfitting. Therefore, our current findings primarily demonstrate the computational and architectural feasibility of secure prediction rather than broad statistical generalizability.

## Methods

### Dataset and preprocessing

For this study, we utilized the publicly available breast cancer spatial transcriptomics dataset from Stahl, et al. ^21^. This dataset consists of 68 Hematoxylin and Eosin images from 23 patients with various breast cancer subtypes, including luminal A, luminal B, triple-negative, HER2 luminal, and HER2 non-luminal. The approximate size of each image is *9500 × 9500*. As our goal was gene expression prediction rather than subtype classification, data from all these subtypes were combined for analysis without distinction. We first performed stain normalization to address potential color variations using the Vahadane technique^39^, which was applied consistently to the entire dataset, encompassing both the training and test sets. As a next step for quality control, we filtered the spots by their read counts. We retained spots with at least 1000 total reads, which resulted in 28,792 spots. This strict quality control focused the evaluation on data points with sufficient signal-to-noise ratios, thereby contributing to the higher performance metrics compared to studies that include lower-coverage spots. Within these spots, we removed genes with a mean expression of zero, leaving 6,216 genes. Among these, the top 250 genes with the highest mean expression across all spots were selected as the target genes to be predicted. These 28,792 spots with 250 genes were then split for model training and testing, with data from patient BC23287 (529 spots) used for testing and data from the remaining 22 patients (28,263 spots) used for training.

Based on the filtered spot locations, we generated corresponding image patches. For privateST model, however, patches were first cropped to $224 \times 224 \times 3{\mathrm{~}}$ pixels and then resized to $64 \times 64 \times 3$ pixels using bilinear interpolation to serve as the final input images. The resized patches were then encrypted for prediction. While the bilinear interpolation from $224 \times 224 \times 3{\mathrm{~}}$ to $64 \times 64 \times 3$ inevitably softens high-frequency fine-grained textures, a qualitative visual inspection confirms that essential tissue architecture—such as general nuclear density and the spatial arrangement of glandular structures—is largely preserved (Supplementary Fig. S3). This retained macroscopic morphology provides the underlying predictive signal for privateST, balancing the need for morphological feature extraction with the slot-capacity constraints of the CKKS encryption scheme. For the comparison models (EfficientNet_b0 and EfficientNet_b4), patches were cropped to $224 \times 224 \times 3$ pixels without resizing, consistent with the original paper^1^.

### The CKKS homomorphic encryption scheme

The Cheon-Kim-Kim-Song (CKKS) scheme is a homomorphic encryption method specifically designed to perform approximate arithmetic on encrypted real numbers^13,40^. This makes it highly suitable for privacy-preserving machine learning applications. Its core principle is to represent data not as exact integers, but as polynomials that can be securely operated upon.

***Encoding and decoding***: The CKKS scheme operates over polynomial rings, not directly on vectors of real numbers. Therefore, a message vector of real numbers must first be encoded into a plaintext polynomial $\:{\upmu\:}\in\:\mathbb{Z}\left[\mathrm{X}\right]/({\mathrm{X}}^{\mathrm{N}}+1)$. This transformation can be achieved using Lagrangian interpolation to find a polynomial that passes through a set of points derived from the message vector. Decoding is the reverse procedure, transforming a decrypted plaintext polynomial back into a vector of approximate real numbers.

***Key generation***: In the symmetric CKKS scheme, a single secret key is used for both encryption and decryption. All keys and cryptographic operations are defined within the polynomial ring $\:{\mathrm{R}}_{\mathrm{q}}={\mathbb{Z}}_{\mathrm{q}}\left[\mathrm{X}\right]/({\mathrm{X}}^{\mathrm{N}}+1)$. The key generation process consists solely of sampling the secret key $\:{\mathrm{s}\in\:\mathrm{R}}_{\mathrm{q}}$ from a distribution of small-norm polynomials.

***Encryption***: A plaintext polynomial $\:{\upmu\:}\in\:\mathbb{Z}\left[\mathrm{X}\right]/({\mathrm{X}}^{\mathrm{N}}+1)$ is encrypted into a ciphertext $\:\mathbf{c}\mathbf{t}=\left({c}_{0},{c}_{1}\right)$ using the secret key $\:\mathbf{s}$. Unlike the public-key variant, encryption involves sampling a uniform random polynomial $\:{\mathrm{a}\in\:\mathrm{R}}_{\mathrm{q}}$ and a small error polynomial $\:\mathrm{e}$ for each encryption. The ciphertext is computed as.

$$\:\mathbf{c}\mathbf{t}=\left({c}_{0},{c}_{1}\right)=\left(-\mathrm{a}\cdot\:\mathrm{s}+{\upmu\:}+\mathrm{e},\mathrm{a}\right)$$

Here, the randomness of $\:\mathrm{a}$ ensures the security of the ciphertext based on the Ring Learning with Errors (RLWE) assumption.

***Decryption***: A ciphertext $\:\mathbf{c}\mathbf{t}=\left({c}_{0},{c}_{1}\right)$ is decrypted using the shared secret key $\:\mathrm{s}$. The computation $\:{\mathrm{c}}_{0}+{\mathrm{c}}_{1}\cdot\:\mathrm{s}$ yields the original plaintext $\:{\upmu\:}$ plus a small error term.

$$\:{c}_{0}+{\mathrm{c}}_{1}\cdot\:\mathrm{s}=\left(-\mathrm{a}\cdot\:\mathrm{s}+{\upmu\:}+\mathrm{e}\right)+\mathrm{a}\cdot\:\mathrm{s}={\upmu\:}+\mathrm{e}.$$

***Addition***: The addition of two ciphertexts, $\:\mathbf{c}\mathbf{t}=\left({c}_{0},{c}_{1}\right)$ from $\:{\upmu\:}$ and $\:\mathbf{c}\mathbf{t}^{\prime\:}=\left({c}_{0}^{\prime\:},{c}_{1}^{\prime\:}\right)$ from $\:{\upmu\:}^{\prime\:}$, is performed element-wise: $\:\mathbf{c}\mathbf{t}+\mathbf{c}{\mathbf{t}}^{{\prime\:}}=({\mathrm{c}}_{0}+{{\mathrm{c}}_{0}}^{{\prime\:}},{\mathrm{c}}_{1}+{\mathrm{c}}_{1}{\prime\:})$. Decrypting the resulting ciphertext yields a value approximately equal to the sum of the original plaintexts, $\:{\upmu\:}+{\upmu\:}{\prime\:}$.

***Constant multiplication***: The multiplication of a ciphertext $\:\mathbf{c}\mathbf{t}=\left({c}_{0},{c}_{1}\right)$ from $\:{\upmu\:}$ by a plaintext constant $\:{{\upmu\:}}^{{\prime\:}}$ is defined as: ${\mathbf{ct}} \cdot { {\mu ^{\prime}}} = \left( {{\mathrm{c}}_{0} \cdot { {\mu ^{\prime}}},{\mathrm{c}}_{1} \cdot { {\mu ^{\prime}}}} \right)$. Decrypting this ciphertext yields an approximation of the product of the plaintexts, ${ {\mu ^{\prime}}} \cdot { {\mu }}$.

***Rotation***: The rotation operation cyclically shifts the slots of the message vector encoded within the plaintext polynomial $\:{\upmu\:}$. Given a ciphertext $\:\mathbf{c}\mathbf{t}=\left({c}_{0},{c}_{1}\right)$ encrypting $\:{\upmu\:}$, a rotation by $\:\mathrm{k}$ positions generate a new ciphertext $\:\mathbf{c}{\mathbf{t}}_{\mathbf{r}\mathbf{o}\mathbf{t}}$ that decrypts to a plaintext polynomial $\:{\upmu\:}$, representing the cyclically shifted vector. Unlike addition and constant multiplication, this operation requires a specialized evaluation key (rotation key) to perform a key-switching procedure.

By leveraging these fundamental operations of homomorphic addition and constant multiplication, a variety of non-linear functions, such as the activation functions in neural networks, can be approximated to enable complex computations on encrypted data. The Orion framework further facilitates this process by automating the complex mapping of neural network operations onto the polynomial arithmetic supported by CKKS^20^. This includes handling non-polynomial activation functions, such as ReLU and softmax. For a detailed mathematical explanation of the ring theory, encoding/decoding, encryption/decryption procedures, and homomorphic operations, please refer to Supplementary Note 2 and Supplementary Fig. S4.

Despite these capabilities, directly deploying standard CNN architectures is impractical primarily because homomorphic rotation is a computationally intensive operation, requiring complex key-switching procedures to maintain security. In CNNs, specifically for strided convolutions, the model must repeatedly shift the entire encrypted data to access neighboring pixels. Since each rotation is computationally expensive, performing this operation frequently creates a major speed bottleneck. Consequently, standard approaches suffer from prohibitive latency, as they not only perform these slow operations repeatedly but also result in sparse data utilization during strided computations. To address these limitations, privateST leverages the single-shot multiplexed packing strategy, which densely packs multiple input channels into a single ciphertext to minimize rotation overhead and maximize computational throughput.

### Experimental model configurations

To systematically assess the impact of these architectural modifications and encryption constraints, we structured the privateST framework into three distinct development phases, as depicted in Fig. 1A:

***ResNet_Base***: The standard ResNet-18 architecture utilizing original ReLU activations and Max Pooling, serving as the plaintext baseline for diagnostic performance.

***ResNet_Approx***: An intermediate configuration that incorporates the arithmetic-compatible modifications (Average Pooling and Polynomial Approximation) but operates on plaintext. This variant allows for quantifying the specific impact of architectural approximations on classification accuracy in isolation from encryption noise.

***ResNet_HE (privateST)***: The final deployment model implemented using the Orion framework. This version integrates all algorithmic and structural optimizations, including single-shot multiplexed packing, to perform fully secure inference on ciphertexts.

### Homomorphic encryption-aware architecture design

Optimizing the ResNet-18 architecture for the ciphertext necessitated a fundamental restructuring to overcome the incompatibility between deep learning operations and homomorphic cryptography constraints. Unlike plaintext implementations, our approach, privateST, is built upon the Orion framework, thereby addressing both arithmetic compatibility—the adaptation of network layers to rely solely on addition and multiplication—and inference latency, the minimization of computational overhead to ensure practical runtime. To systematically achieve these dual objectives, we implemented two strategic adaptations as illustrated in Fig. 1.

1. Ensuring arithmetic compatibility via linearization and approximation.

Standard Convolutional Neural Networks (CNNs) rely heavily on non-linear operations that involve value comparisons. However, the CKKS scheme is inherently limited to arithmetic operations (addition and multiplication) and cannot perform direct comparisons on encrypted data. To resolve this incompatibility without sacrificing feature extraction capabilities, we re-engineered two critical components of the network (Fig. 1B and C).

*Substitution of pooling layers (linearization)*: We replaced the standard Max Pooling layers with Average Pooling. Unlike Max Pooling, which relies on the non-linear ${\mathrm{max}}\left( {{\mathrm{a}},{\mathrm{b}}} \right)$ operator, Average Pooling over a *k × k* window is strictly a linear transformation defined as.

$${y_{i,j}}=\frac{1}{{{k^2}}}\mathop \sum \limits_{{m=0}}^{{k - 1}} \mathop \sum \limits_{{n=0}}^{{k - 1}} {x_{i+m,~j+n}}$$

Since this operation consists solely of homomorphic additions and scalar multiplications (by factor $1/{k^2}$), it is natively supported by the CKKS scheme and avoids computationally expensive comparisons.

*Approximation of activation functions*: We replaced the ReLU activation function with a high-precision polynomial approximation. The ReLU function is defined as $ReLU\left( x \right)={\mathrm{max}}\left( {x,0} \right)$, which, like Max Pooling, is a comparison-based function that cannot be directly evaluated on encrypted ciphertexts. To achieve accurate non-linearity within the encrypted domain, we adopted a minimax composite polynomial approach. The approximate activation function, P(x), is formulated as a composition of three polynomials.

$$P\left( x \right)~=~{h_{27}}\left( {{g_{15}}\left( {{f_{15}}\left( x \right)} \right)} \right)$$

where ${{\mathrm{f}}_{15}},~{g_{15}}{\mathrm{~and~}}{{\mathrm{h}}_{27}}$, denote minimax polynomials of degrees 15, 15, and 27, respectively. This composite structure allows for a high-degree approximation while minimizing the multiplicative depth required for homomorphic evaluation.

To ensure the numerical stability of this high-degree composite approximation, polynomial inputs are constrained to remain within the valid approximation range. Every polynomial activation in our architecture is preceded by a BatchNorm layer, which normalizes its input distribution to approximately zero mean and unit variance.

2. Maximizing throughput via structural and algorithmic optimizations

Even with an arithmetic-compatible architecture, direct implementation of CNNs suffers from prohibitive latency. To address this, privateST employs a comprehensive optimization strategy targeting two critical sources of overhead:

*Limited multiplicative levels*: In the CKKS scheme, consuming levels necessitates computationally expensive bootstrapping to refresh the ciphertext. Thus, minimizing the depth of operations is paramount.

*High cost of rotations*: Even within available levels, homomorphic rotations are inherently slow due to complex ‘key-switching’ procedures. Therefore, reducing the sheer number of rotation operations is equally critical for accelerating inference.

With these dual objectives, privateST implements the following optimizations:

*Structural optimization (single-shot multiplexed packing)*: To maximize data density, privateST adopts the single-shot multiplexed packing strategy as the fundamental data layout for all convolutional layers. As visually depicted in Fig. 1D, privateST employs ‘multiplexed packing,’ where multiple input channels are densely packed into a single ciphertext grid.

- In standard convolutions (Stride = 1): This dense interleaved layout allows standard convolutions to be executed efficiently without modifying the data structure.
- In strided convolutions (Stride > 1): The primary advantage of this layout emerges in strided layers (stride = 2), where the filter skips pixels to reduce spatial dimensions. While standard packing would leave these skipped spaces empty (zeros), creating sparse computation, the multiplexed layout ensures these spaces are already occupied by data from other channels. Crucially, this pre-aligned density enables the convolution to be executed in a ‘single shot’—consuming only one multiplicative level—by eliminating the need for a secondary ‘mask-and-collect’ step to remove invalid data.

*Algorithmic optimization*: With the data densely packed across all layers, the remaining challenge is the rotation complexity, which scales linearly with the filter size ($O\left( f \right))$. To mitigate this, privateST leverages the Baby-Step Giant-Step (BSGS) algorithm. By decomposing the rotations, this optimization reduces the complexity to square root time.

$${C_{rot}} \approx O\left( {\sqrt f } \right)$$

This significant reduction in algorithmic complexity minimizes the total number of expensive key-switching operations required for feature extraction.

### Comparison models

BrSTNet^1^ is a deep learning-based framework designed to predict spatial gene expression profiles directly from $24 \times 224 \times 3$ H&E-stained histology image patches. By effectively capturing complex morphological features associated with transcriptional variations, BrSTNet elucidates the underlying association between tissue morphology and gene expression, offering a cost-effective alternative to traditional spatial transcriptomics technologies.

To evaluate predictive accuracy, we benchmarked privateST against the top-performing architectures from the BrSTNet framework: EfficientNet_b0 and EfficientNet_b4. EfficientNet is a family of convolutional neural networks that optimizes both accuracy and efficiency by employing a compound scaling method, which uniformly scales network width, depth, and resolution. In this study, we selected EfficientNet_b0 as a lightweight baseline and EfficientNet_b4 as a more complex architecture capable of capturing finer morphological details, representing a range of model capacities.

For a fair comparison, we replicated the exact experimental setup described in the original BrSTNet study. This included adopting the auxiliary learning structure, setting the loss weight to 40, and utilizing all remaining genes for the auxiliary task.

### Gene expression normalization

Gene expression values underwent a two-step normalization process to ensure numerical stability and consistent scaling. First, raw read counts (*x*) were log-transformed to reduce skewness and stabilize variance:

$$x'~=~log\left( {x~+~1} \right)$$

Second, these log-transformed values were standardized (z-scored) on a per-gene basis. The normalized expression value (*z*) is calculated as:

$$z=\left( {x^{\prime} - {\mu _{train}}} \right)/{\sigma _{train}}$$

Crucially, the mean (${\mu _{train}}$) and standard deviation (${\sigma _{train}}$) statistics were derived from the training set and subsequently applied to normalize the test set. This strict separation prevents data leakage and ensures that the evaluation reflects a realistic clinical scenario where test data statistics are unknown.

Since the normalization parameters (${\mu _{train}},~{\sigma _{train}}$) are derived from the training set, a high normalized value in the test set indicates that the gene’s expression level is relatively higher than its average expression in the training distribution. Consequently, the “top-ranked genes” essentially represent targets with the strongest biological signals. These high-signal genes are prioritized because they are more likely to exhibit distinct morphological signatures in H&E images, making them inherently more predictable^2^. In contrast, low-expression genes are often dominated by stochastic noise and lack clear visual correlates, posing a greater challenge for image-based prediction models.

### Evaluation metrics

To assess the performance of gene expression prediction, we utilized three metrics: Pearson Correlation Coefficient (PCC), Spearman Correlation Coefficient (SCC), and Root Mean Square Error (RMSE). PCC measures the linear correlation between the predicted and observed expression profiles, indicating how well the model captures the gene expression patterns:

$${\mathrm{PCC}}=\frac{{\mathop \sum \nolimits_{{i=1}}^{N} \left( {{y_i} - \bar {y}} \right)\left( {{{\hat {y}}_i} - \overline {{\hat {y}}} } \right)}}{{\sqrt {\mathop \sum \nolimits_{{i=1}}^{N} {{\left( {{y_i} - \bar {y}} \right)}^2}} \sqrt {\mathop \sum \nolimits_{{i=1}}^{N} {{\left( {{y_i} - \hat {y}} \right)}^2}} }}$$

SCC measures the monotonic correlation between the predicted and observed expression profiles based on their ranks. Unlike PCC, SCC assesses how well the model preserves the relative ordering of gene expression levels, offering robustness against outliers and non-linear associations:

$$SCC=1 - ~\frac{{6\mathop \sum \nolimits_{{i=1}}^{N} d_{i}^{2}}}{{N\left( {{N^2} - 1} \right)}}$$

where ${d_i}$ represents the difference between the ranks of the ground truth and the predicted values.

RMSE measures the standard deviation of the prediction errors, penalizing larger deviations in expression values more heavily:

$${\mathrm{RMSE}}=\sqrt {\frac{1}{N}\mathop \sum \limits_{{i=1}}^{N} {{\left( {{y_i} - {{\hat {y}}_i}} \right)}^2}}$$

## Supplementary Information

Below is the link to the electronic supplementary material.

Supplementary Material 1

Supplementary Material 2

Supplementary Material 3

## Data Availability

This study utilizes an existing public dataset. The breast cancer spatial transcriptomics dataset is available at https://data.mendeley.com/datasets/29ntw7sh4r/5. All original code has been deposited at https://github.com/2w21234/privateST and is publicly available.

## Declarartions

### Use of generative AI

During the preparation of this work the authors used Gemini in order to refine the wording. After using this tool, the authors reviewed and edited the content as needed and take full responsibility for the content of the publication.
