# Supplementary material for: privateST: a feasible framework for privacy-preserving spatial transcriptomics prediction from histopathology images
Source: Sci Rep. 2026 Jun 3;16:25330. doi: 10.1038/s41598-026-55961-4 (PMC13473608; doi:10.1038/s41598-026-55961-4)
Supplement: Supplementary file 1 [file 41598_2026_55961_MOESM1_ESM.docx]

**privateST: A Feasible Framework for Privacy-Preserving Spatial Transcriptomics Prediction from Histopathology Images**

**Hakin Kim^1^, Miran Kim^2,*^, Buhm Han^1,3,4,*^**

^1^ Interdisciplinary Program in Bioengineering, Seoul National University, Seoul, Republic of Korea

^2^ Department of Mathematics, Hanyang University, Seoul, Republic of Korea

^3^ Convergence Dementia Research Center, Seoul National University Medical Research Center, Seoul, Republic of Korea

^4^ Department of Biomedical Sciences, BK21 Plus Biomedical Science Project, Seoul National University College of Medicine, Seoul, Republic of Korea

**Supplementary materials**

**Supplementary Note 1**

**Detailed analysis based on Pearson correlation coefficient**

To complement the benchmarking results in **Figure 4**, we conducted a more detailed evaluation centered on the Pearson correlation coefficient (PCC). While **Figure 4** compared overall performance across different gene sets, here we re-examine the same dataset with a specific focus on PCC to highlight gene-level predictive differences among privateST, EfficientNet_b0, and EfficientNet_b4 (**Supplementary Figure 2**). **Figure 4a** presents the results for the overall average and for the top 10 best-predicted genes, as ranked by the privateST model's PCC. privateST achieved the highest overall average PCC of 0.673, outperforming both EfficientNet_b0 (0.656) and EfficientNet_b4 (0.626). Its advantage was particularly pronounced for the gene H3-3B. For *H3-3B*, privateST achieved a PCC of 0.649, substantially outperforming both EfficientNet_b0 (0.569) and EfficientNet_b4 (0.547). Notably, *H3-3B* is a histone H3.3 variant that has been shown to be significantly overexpressed in breast cancer tissues compared to adjacent normal tissues, and it plays a role in tumor progression and metastasis through epigenetic regulation and autophagy pathways ^1-3^.

**Figure 4b** presents the PCC distribution across all genes. privateST achieved the largest number of genes with $PCC \geq0.5$ (23 genes), whereas EfficientNet_b0 and EfficientNet_b4 reached 18 and 16 genes, respectively. In addition, privateST yielded the fewest negatively correlated predictions (11 genes), compared to 16 for EfficientNet_b0 and 13 for EfficientNet_b4.

**Supplementary Note 2**

Below we describe the detailed mathematical formulas for the CKKS homomorphic encryption scheme utilized in privateST. The overall workflow is illustrated in **Supplementary Figure 3**.

**Ring**

A ring $R$ is a set with two binary operations, addition ($+$) and multiplication ($\cdot$), satisfying following axioms^4,5^.

1. $R$ is an abelian group, which means that it satisfies the following five conditions:

- $R$ is closed under addition:

$$a+b \in R for all a, b in R$$

- $R$ is associative under addition:

$$\left( a+b \right)+c=a+\left( b+c \right) for all a, b, c in R$$

- $R$ is commutative under addition:

$$a+b=b+a for all a, b in R$$

- Additive identity exists in $R$:

$$There exists an element 0 in R such that a+ 0= 0+a=a for all a in R$$

- Additive inverse exists in $R$:

$$For each a in R, there exists -a in R such that a+\left( -a \right)=0$$

2. $R$ is associative under multiplication:

$$\left( a\cdot b \right)\cdot c=a\cdot\left( b\cdot c \right) for all a,b,c in R$$

3. $R$ is distributive with respect to addition

$$a\cdot\left( b+c \right)=\left( a\cdot b \right)+\left( a\cdot c \right)\mathrm{and}\left( b+c \right)\cdot a=\left( b\cdot a \right)+\left( c\cdot a \right) for all a, b, c in R$$

For example, $\mathbb{Z}$ (the integer set) is the representative ring in mathematics. Also, the set of integers modulo p, $\mathbb{Z}_{p}=\left\{ 0,1,2,\cdots,p-1 \right\}$, is also a ring satisfying the conditions above. Specifically, $\mathbb{Z}_{p}$ is called the residue ring of $\mathbb{Z}$ modulo an integer p. If p=4, then $\mathbb{Z}_{4}=\left\{ 0,1,2,3 \right\}$, that is, we regard 4 as 0 in this ring. Also, $2+3\equiv5\equiv1 \left( mod 4 \right)\in\mathbb{Z}_{4}$, $2\cdot3\equiv6\equiv2 \left( mod 4 \right)\in\mathbb{Z}_{4}$ and $-1\equiv3 (mod 4$). $\mathbb{Z}$ and $\mathbb{Z}_{p}$ are also commutative under multiplication: $b\cdot a=a\cdot b for all a,b in R$.

**Homomorphism and isomorphism**

Let $\emptyset$ to be a map from a ring $R$ to a ring $R'$. If $\emptyset\left( a+b \right)=\emptyset\left( a \right)+\emptyset\left( b \right)$ and $\emptyset\left( a\cdot b \right)=\emptyset\left( a \right)\cdot\emptyset\left( b \right)$ for all $a, b in R$, then the map $\emptyset$ is homomorphic. Homomorphism is a key idea in HE. This property enables privateST to ensure the protection against attacks to hack users’ Hematoxylin and Eosin image data, because users send $\emptyset\left( x \right)$ which is encrypted form instead of $x$(the image data) where $\emptyset$ means encryption step. Since we add some errors to enhance the security, $\emptyset\left( a+b \right)\approx\emptyset\left( a \right)+\emptyset\left( b \right)$ and $\emptyset\left( a\cdot b \right)\approx\emptyset\left( a \right)\cdot\emptyset\left( b \right)$. If a homomorphic map preserves one-to-one (if there exists an inverse map for each map), the map is called isomorphic.

**Polynomial ring and its quotients**

Let $R$ be a commutative ring and $x$ be a variable. Then the polynomial ring $R[x]$ is defined to be the set consisting of the following elements:

$$a_{0}+a_{1}x+\cdots+a_{n}x^{N}=\sum_{i=0}^{N} a_{i}x^{i}\mathrm{where}a_{i}\in R$$

Specifically, privateST is based on quotients rings of polynomial rings ${\mathbb{Z}_{p}/(x}^{N}+1$) where N is a ring dimension and is power of two. In case of p=4 and N=2, ${\mathbb{Z}_{4}/(x}^{2}+1)$, $\left( 1+3x \right)+\left( 1+2x \right)\equiv2+5x\equiv2+x mod \left( x^{2}+1 \right)$ because we regard $x^{2}+1$ as 0 (the additive identity) in ${\mathbb{Z}_{4}/(x}^{2}+1$). Also, $\left( 1+3x \right)\left( 1+2x \right)\equiv1+5x+6x^{2}\equiv1+5x+6x^{2}\equiv1+5x+6\left( x^{2}+1 \right)+\left( -6 \right)\equiv1+5x+6\left( x^{2}+1 \right)+\left( -6 \right)\equiv1+5x+2\equiv3+5x mod (x^{2}+1)$ where we omitted the multiplication symbol “$\cdot$” for convenience.

HE is recognized as promising field for dealing with privacy issues. For privateST, we applied CKKS scheme which is one of the most well-known homomorphic encryption methods. Below we describe the mathematical steps of privateST.

**Encoding and decoding of CKKS**

Let $\mathbf{m}=(m_{1}, m_{2},\cdots, m_{N/2})\in\mathbb{C}^{N/2}$be a message vector which is image pixel data. Since CKKS is based on a polynomial space, $R_{q}^{2}$, the message vector $m$ should be isomorphically mapped into a polynomial form $\mu\left( X \right)=\sum_{i=0}^{N-1} a_{i}X^{i}$. The encoding procedure consists of three steps:

1. Since $m$ is a complex vector, we expand $m$ to $\mathbf{m'}$=($m_{1}, m_{2},\cdots, m_{N/2},\bar{m_{1}}, \bar{m_{2}},\cdots, \bar{m_{N/2}}$)$\in\mathbb{C}^{N}$ by adding conjugate elements where $\bar{a+bi}=a-bi$ for all a, b in $\mathbb{R}$ and let $\mathbf{m}^{\mathbf{'}}=$($m_{1}, m_{2},\cdots, m_{N/2},\bar{m_{N/2}}, \bar{m_{N/2-1}},\cdots, \bar{m_{1}})=(z_{1},z_{2},\cdots,z_{N})$ for convenience.

2. It is known that if N is power of two, the equation $X^{N}+1=0$ has roots $\{\xi, \xi^{3}, \xi^{5},\cdots, \xi^{2N-1}\}$ where $\xi=e^{\pi i/N}$ and $i=\sqrt{-1}$. Then we can get a (N-1)th order polynomial that passes a set of points {($\xi$, $z_{1}$),($\xi^{3}$, $z_{2}$),$\cdots$,($\xi^{2N-1}$, $z_{N}$)} satisfying $\mu_{1}\left( x \right)=\sum_{i=0}^{N-1} \alpha_{i}x^{i}=z_{j} for j in \{1, \ldots, N\}$ with roots $\{\xi, \xi^{3}, \xi^{5},\cdots, \xi^{2N-1}\}$, that is, $\boldsymbol{A\alpha}=\mathbf{z}$where $\mathbf{A}=\left[ \begin{matrix} 1 & \xi& \xi^{2} & \cdots& \xi^{N-1} \\ 1 & \xi^{3} & \left( \xi^{3} \right)^{2} & \cdots& \left( \xi^{3} \right)^{N-1} \\ \vdots& \vdots& \vdots& \ddots& \vdots\\ 1 & \xi^{2N-1} & {{(\xi}^{2N-1})}^{2} & \cdots& {{(\xi}^{2N-1})}^{N-1} \end{matrix} \right]$ and $\boldsymbol{\alpha}=\left[ \begin{matrix} \alpha_{0} \\ \alpha_{1} \\ \vdots\\ \alpha_{N-1} \end{matrix} \right]$ is a vector of coefficients. Therefore, we have $\boldsymbol{\alpha=}\mathbf{A}^{\mathbf{-1}}\mathbf{z}$. But if we have lots of points (have large N), calculating $\mathbf{A}^{\mathbf{-1}}$ is computationally hard. So, we may apply an alternative method: the Lagrange interpolation, such that $\mu_{1}\left( x \right)=\sum_{i=1}^{N} z_{i}l_{i}\left( x \right)\mathbb{\in R[}X]/(X^{N}+1) where l_{i}\left( x \right)=\frac{\prod_{j\neq i} (x-\xi^{2j-1})}{\prod_{j\neq i} (\xi^{2i-1}-\xi^{2j-1})}$.

3. After applying the Lagrange interpolation, since we need to encode${\boldsymbol{m}\mathbb{\in C}}^{N/2}$ not into a ring $\mathbb{R[}X]/(X^{N}+1)$ but into a ring $R=\mathbb{Z[}X]/(X^{N}+1)$, we have to round off each real coefficient of $\mu_{1}\left( X \right)$ to the nearest integer, allowing negligible error. A parameter related to this error is scaling factor $\Delta$. Because we round off the coefficients of $\mu_{1}\left( X \right)\cdot\Delta$ instead of $\mu_{1}\left( X \right)$, as the scaling factor $\Delta$ increases, the precision increases. For example, if N=4, $\boldsymbol{m}=(0,1)\in\mathbb{C}^{2}$, then$\mu_{1}\left( x \right)=\frac{1}{2}-\frac{1}{2\sqrt{2}}x+\frac{1}{2\sqrt{2}}x^{3}$ that passes {$(\xi,0)$, $\left( \xi^{3},1 \right),$ $\left( \xi^{5},1 \right),$ $(\xi^{7},0)$}. Suppose we try two different scaling factors, $\Delta_{1}=10$ and $\Delta_{2}=100$. After rounding off step, $\left\lfloor\mu_{1}\left( x \right)\cdot\Delta_{1} \right\rceil=5-4x+4x^{30}$ and $\left\lfloor\mu_{1}\left( x \right)\cdot\Delta_{2} \right\rceil=50-35x+35x^{3}$. Although $\left\lfloor\mu_{1}\left( x \right)\cdot\Delta_{1} \right\rceil\approx\mu_{1}\left( x \right)\cdot\Delta_{1}$ and $\left\lfloor\mu_{1}\left( x \right)\cdot\Delta_{2} \right\rceil\approx\mu_{1}\left( x \right)\cdot\Delta_{2}$, the precision is better in the case of $\Delta_{2}$. This means that by using a sufficiently large scaling factor, we can reduce the errors to a negligible level. Given a scaling factor $\Delta$, $\mu\left( X \right)= \left\lfloor\mu_{1}\left( x \right)\cdot\Delta\right\rceil\mathbb{\in Z[}X]/(X^{N}+1)$ is called plaintext that is encoded from a message vector.

The decoding procedure is a reverse of encoding procedure. Given a plaintext $\mu\left( X \right)$, by plugging in $\{\xi, \xi^{5},\cdots, \xi^{2N-3}\}$ for X, ${(m}_{1}, m_{2},\cdots, m_{N/2})$ can be approximately calculated where $\xi=e^{\pi i/4}$ . The precision in decoding also depends on the scaling factor $\Delta$. In case of $\Delta_{1}$ and $\Delta_{2}$ from the above examples, $\left( \left\lfloor\mu_{1}\left( \xi\right)\cdot\Delta_{1} \right\rceil,\left\lfloor\mu_{1}\left( \xi^{5} \right)\cdot\Delta_{1} \right\rceil\right)\approx\left( -0.0657\cdot\Delta_{1},1.0657\cdot\Delta_{1} \right)$ and$(\left\lfloor\mu_{1}\left( \xi\right)\cdot\Delta_{2} \right\rceil,\left\lfloor\mu_{1}\left( \xi^{5} \right)\cdot\Delta_{2} \right\rceil)\approx\left( 0.0503\cdot\Delta_{2},0.9950\cdot\Delta_{2} \right)$. Therefore, the error is smaller in the case of $\Delta_{2}$ since |$0-(-0.0657)|>|0-0.0503|$ and $\left| 1-1.0657 \right|>|1-0.9950|$. We note that even though there exists an error during the encoding and decoding procedures, the error is ignorable and does not affect the security.

**Encryption and decryption of CKKS**

In encryption procedure, we transform the plaintext data into a ciphertext with a public key, while in decryption procedure we transform the ciphertext data into a plaintext data. We define a ring $R_{q}=\mathbb{Z}_{q}[X]/(X^{N}+1)$ for convenience. To encrypt a plaintext $\mu\left( X \right)\in R$, we first generate a secret key $s\in R_{q}$ of which coefficients are sampled from $\{-1,0,1\}$ with an integral hamming weight $h$, which means that the number of coefficients equal to zero is ‘$h$’. We generate noisy polynomials $e_{1}, e_{2}$ and $e_{3}$ in $R_{q}$ of which coefficients are sampled from a discrete Gaussian distribution, with standard deviation of 3.2 in our case. And we sample $a\in R_{q}$ uniformly at random. Then we calculate $b=e_{1}-a\cdot s$ which becomes an element of a public key $\mathbf{p}=(b,a)$. For encryption step, we additionally sample $v\in R_{q}$ of which coefficients are sampled from a set $\{-1,0,1\}$ with probability 0.25 for each of -1 and 1 and 0.5 for 0. Then, with a generated public key $p$, we encrypt $\mu\left( X \right)$ into $ct=v\cdot p+\left( \mu+e_{2}, e_{3} \right)=\left( v\cdot b+\mu+e_{2},v\cdot a+e_{3} \right) \in R_{q}^{2}$. If we decrypt a ciphertext $\mathbf{ct}=(c0,c1)$ into a plaintext, $c0+c1\cdot s$ approximately returns the original plaintext including a noise. For example, if $(c0,c1)=\left( v\cdot b+\mu+e_{2},v\cdot a+e_{3} \right)$ then $v\cdot b+\mu+e_{2}+\left( v\cdot a+e_{3} \right)\cdot s=\mu+v\cdot\left( b+a\cdot s \right)+e_{2}+e_{3}=\mu+v\cdot e_{1}+e_{2}+e_{3}=\mu+e where e=v\cdot e_{1}+e_{2}+e_{3}$. Since the noise $e$ is small enough, it does not affect the overall results.

**Homomorphic operations in CKKS**

The CKKS homomorphic encryption scheme enables meaningful computations to be performed directly on encrypted data. Two of the core supported operations are constant multiplication, for multiplying an encrypted value by an unencrypted plaintext value, and addition, for combining multiple encrypted values.

1. Addition

Let $\mathbf{ct}=\left( c0,c1 \right)=\left( v\cdot b+\mu+e_{2},v\cdot a+e_{3} \right)$ and $\mathbf{ct'}=\left( c0',c1' \right)=\left( v'\cdot b'+\mu'+e_{2}',v'\cdot a'+e_{3}' \right)$ with sharing a same secret key $s$ and the scaling factor $\Delta$ should be same to get a meaningful result. We simply define addition: $\mathbf{ct}_{\mathbf{add}}=\mathbf{ct}+\mathbf{c}\mathbf{t}^{\mathbf{'}}=(c0+c0^{'},c1+c1')$, that is, elementwise addition. Then when decrypting $\mathbf{ct+c}\mathbf{t}^{\mathbf{'}}$, $\left( c0+c0^{'} \right)+\left( c1+c1^{'} \right)\cdot s=\left( c0^{'}+c1^{'}\cdot s \right)+\left( c0^{'}+c1^{'}\cdot s \right)=\left( \mu+\mu^{'} \right)+\left( e+e^{'} \right)\approx\mu’+\mu$, which means the plaintext after addition is bounded by the sum of two small errors.

2. Constant Multiplication

Let $\mathbf{ct}=\left( c0,c1 \right)=\left( v\cdot b+\mu+e_{2},v\cdot a+e_{3} \right)$ and $\mu' \in R$ with scaling factor $\Delta$. Then we define constant multiplication as $\mathbf{ct}_{\mathbf{cMult}}=\left( c0\cdot\mu’,c1\cdot\mu’ \right)\in R_{q}^{2}$ with scaling factor $\Delta^{2}$. Then in the decryption step, $c0\cdot\mu’+\left( c1\cdot\mu’ \right)\cdot s=\mu’\cdot\left( c0+c1\cdot s \right)= \mu’\cdot(\mu+e) \approx\mu’\cdot\mu$.

After a multiplication, the scaling factor increases to $\Delta^{2}$ from $\Delta$, which increases the computational burden since the significant number increases exponentially as the number of multiplications increases. So, if we divide the scaling factor $\Delta^{2}$ by $\Delta^{-1}$, we maintain the scaling factor. Given a ciphertext $\mathbf{ct}=\left( c0,c1 \right)$, the ciphertext after rescaling is $\mathbf{c}\mathbf{t}^{\mathbf{'}}=\left\lfloor\mathbf{ct}\cdot\Delta^{-1} \right\rceil$ $({mod \Delta}^{-1}q)$ of which plaintext is equivalent to $\left\lfloor c0\cdot\Delta^{-1} \right\rceil+\left\lfloor c1\cdot\Delta^{-1} \right\rceil\cdot s\approx(c0+c1\cdot s)\cdot\Delta^{-1}$.

**Supplementary Note 3**

**ResNet18-Base**

ResNet(

(conv1): Conv2d(3, 64, kernel_size=(7, 7), stride=(2, 2), padding=(3, 3), bias=False)

(bn1): BatchNorm2d(64, eps=1e-05, momentum=0.1, affine=True, track_running_stats=True)

(relu): ReLU(inplace=True)

(maxpool): MaxPool2d(kernel_size=3, stride=2, padding=1, dilation=1, ceil_mode=False)

(layer1): Sequential(

(0): BasicBlock(

(conv1): Conv2d(64, 64, kernel_size=(3, 3), stride=(1, 1), padding=(1, 1), bias=False)

(bn1): BatchNorm2d(64, eps=1e-05, momentum=0.1, affine=True, track_running_stats=True)

(relu): ReLU(inplace=True)

(conv2): Conv2d(64, 64, kernel_size=(3, 3), stride=(1, 1), padding=(1, 1), bias=False)

(bn2): BatchNorm2d(64, eps=1e-05, momentum=0.1, affine=True, track_running_stats=True)

)

(1): BasicBlock(

(conv1): Conv2d(64, 64, kernel_size=(3, 3), stride=(1, 1), padding=(1, 1), bias=False)

(bn1): BatchNorm2d(64, eps=1e-05, momentum=0.1, affine=True, track_running_stats=True)

(relu): ReLU(inplace=True)

(conv2): Conv2d(64, 64, kernel_size=(3, 3), stride=(1, 1), padding=(1, 1), bias=False)

(bn2): BatchNorm2d(64, eps=1e-05, momentum=0.1, affine=True, track_running_stats=True)

)

)

(layer2): Sequential(

(0): BasicBlock(

(conv1): Conv2d(64, 128, kernel_size=(3, 3), stride=(2, 2), padding=(1, 1), bias=False)

(bn1): BatchNorm2d(128, eps=1e-05, momentum=0.1, affine=True, track_running_stats=True)

(relu): ReLU(inplace=True)

(conv2): Conv2d(128, 128, kernel_size=(3, 3), stride=(1, 1), padding=(1, 1), bias=False)

(bn2): BatchNorm2d(128, eps=1e-05, momentum=0.1, affine=True, track_running_stats=True)

(downsample): Sequential(

(0): Conv2d(64, 128, kernel_size=(1, 1), stride=(2, 2), bias=False)

(1): BatchNorm2d(128, eps=1e-05, momentum=0.1, affine=True, track_running_stats=True)

)

)

(1): BasicBlock(

(conv1): Conv2d(128, 128, kernel_size=(3, 3), stride=(1, 1), padding=(1, 1), bias=False)

(bn1): BatchNorm2d(128, eps=1e-05, momentum=0.1, affine=True, track_running_stats=True)

(relu): ReLU(inplace=True)

(conv2): Conv2d(128, 128, kernel_size=(3, 3), stride=(1, 1), padding=(1, 1), bias=False)

(bn2): BatchNorm2d(128, eps=1e-05, momentum=0.1, affine=True, track_running_stats=True)

)

)

(layer3): Sequential(

(0): BasicBlock(

(conv1): Conv2d(128, 256, kernel_size=(3, 3), stride=(2, 2), padding=(1, 1), bias=False)

(bn1): BatchNorm2d(256, eps=1e-05, momentum=0.1, affine=True, track_running_stats=True)

(relu): ReLU(inplace=True)

(conv2): Conv2d(256, 256, kernel_size=(3, 3), stride=(1, 1), padding=(1, 1), bias=False)

(bn2): BatchNorm2d(256, eps=1e-05, momentum=0.1, affine=True, track_running_stats=True)

(downsample): Sequential(

(0): Conv2d(128, 256, kernel_size=(1, 1), stride=(2, 2), bias=False)

(1): BatchNorm2d(256, eps=1e-05, momentum=0.1, affine=True, track_running_stats=True)

)

)

(1): BasicBlock(

(conv1): Conv2d(256, 256, kernel_size=(3, 3), stride=(1, 1), padding=(1, 1), bias=False)

(bn1): BatchNorm2d(256, eps=1e-05, momentum=0.1, affine=True, track_running_stats=True)

(relu): ReLU(inplace=True)

(conv2): Conv2d(256, 256, kernel_size=(3, 3), stride=(1, 1), padding=(1, 1), bias=False)

(bn2): BatchNorm2d(256, eps=1e-05, momentum=0.1, affine=True, track_running_stats=True)

)

)

(layer4): Sequential(

(0): BasicBlock(

(conv1): Conv2d(256, 512, kernel_size=(3, 3), stride=(2, 2), padding=(1, 1), bias=False)

(bn1): BatchNorm2d(512, eps=1e-05, momentum=0.1, affine=True, track_running_stats=True)

(relu): ReLU(inplace=True)

(conv2): Conv2d(512, 512, kernel_size=(3, 3), stride=(1, 1), padding=(1, 1), bias=False)

(bn2): BatchNorm2d(512, eps=1e-05, momentum=0.1, affine=True, track_running_stats=True)

(downsample): Sequential(

(0): Conv2d(256, 512, kernel_size=(1, 1), stride=(2, 2), bias=False)

(1): BatchNorm2d(512, eps=1e-05, momentum=0.1, affine=True, track_running_stats=True)

)

)

(1): BasicBlock(

(conv1): Conv2d(512, 512, kernel_size=(3, 3), stride=(1, 1), padding=(1, 1), bias=False)

(bn1): BatchNorm2d(512, eps=1e-05, momentum=0.1, affine=True, track_running_stats=True)

(relu): ReLU(inplace=True)

(conv2): Conv2d(512, 512, kernel_size=(3, 3), stride=(1, 1), padding=(1, 1), bias=False)

(bn2): BatchNorm2d(512, eps=1e-05, momentum=0.1, affine=True, track_running_stats=True)

)

)

(avgpool): AdaptiveAvgPool2d(output_size=(1, 1))

(fc): Linear(in_features=512, out_features=250, bias=True)

)

**Approx_ST & Approx_HE (privateST)**

ResNet(

level=None

(conv1): Conv2d(in_channels=3, out_channels=64, kernel_size=(7, 7), stride=(2, 2), padding=(3, 3), dilation=(1, 1), groups=1, level=None, bsgs_ratio=2.0)

(bn1): BatchNorm2d(level=None, level=None, fused=False)

(act): ReLU(

level=None, degrees=[15, 15, 27]

(sign): _Sign(

level=None, degrees=[15, 15, 27]

(mult): Mult(level=None)

(acts): Sequential(

(0): Chebyshev(level=None, degree=15)

(1): Chebyshev(level=None, degree=15)

(2): Chebyshev(level=None, degree=27)

)

)

(mult1): Mult(level=None)

(mult2): Mult(level=None)

)

(First_Avgpool): AvgPool2d(AvgPool2d(kernel_size=(3, 3), stride=(2, 2) level = None))

(layers): ModuleList(

(0): Sequential(

(0): BasicBlock(

level=None

(conv1): Conv2d(in_channels=64, out_channels=64, kernel_size=(3, 3), stride=(1, 1), padding=(1, 1), dilation=(1, 1), groups=1, level=None, bsgs_ratio=2.0)

(bn1): BatchNorm2d(level=None, level=None, fused=False)

(act1): ReLU(

level=None, degrees=[15, 15, 27]

(sign): _Sign(

level=None, degrees=[15, 15, 27]

(mult): Mult(level=None)

(acts): Sequential(

(0): Chebyshev(level=None, degree=15)

(1): Chebyshev(level=None, degree=15)

(2): Chebyshev(level=None, degree=27)

)

)

(mult1): Mult(level=None)

(mult2): Mult(level=None)

)

(conv2): Conv2d(in_channels=64, out_channels=64, kernel_size=(3, 3), stride=(1, 1), padding=(1, 1), dilation=(1, 1), groups=1, level=None, bsgs_ratio=2.0)

(bn2): BatchNorm2d(level=None, level=None, fused=False)

(act2): ReLU(

level=None, degrees=[15, 15, 27]

(sign): _Sign(

level=None, degrees=[15, 15, 27]

(mult): Mult(level=None)

(acts): Sequential(

(0): Chebyshev(level=None, degree=15)

(1): Chebyshev(level=None, degree=15)

(2): Chebyshev(level=None, degree=27)

)

)

(mult1): Mult(level=None)

(mult2): Mult(level=None)

)

(add): Add(level=None)

(shortcut): Sequential()

)

(1): BasicBlock(

level=None

(conv1): Conv2d(in_channels=64, out_channels=64, kernel_size=(3, 3), stride=(1, 1), padding=(1, 1), dilation=(1, 1), groups=1, level=None, bsgs_ratio=2.0)

(bn1): BatchNorm2d(level=None, level=None, fused=False)

(act1): ReLU(

level=None, degrees=[15, 15, 27]

(sign): _Sign(

level=None, degrees=[15, 15, 27]

(mult): Mult(level=None)

(acts): Sequential(

(0): Chebyshev(level=None, degree=15)

(1): Chebyshev(level=None, degree=15)

(2): Chebyshev(level=None, degree=27)

)

)

(mult1): Mult(level=None)

(mult2): Mult(level=None)

)

(conv2): Conv2d(in_channels=64, out_channels=64, kernel_size=(3, 3), stride=(1, 1), padding=(1, 1), dilation=(1, 1), groups=1, level=None, bsgs_ratio=2.0)

(bn2): BatchNorm2d(level=None, level=None, fused=False)

(act2): ReLU(

level=None, degrees=[15, 15, 27]

(sign): _Sign(

level=None, degrees=[15, 15, 27]

(mult): Mult(level=None)

(acts): Sequential(

(0): Chebyshev(level=None, degree=15)

(1): Chebyshev(level=None, degree=15)

(2): Chebyshev(level=None, degree=27)

)

)

(mult1): Mult(level=None)

(mult2): Mult(level=None)

)

(add): Add(level=None)

(shortcut): Sequential()

)

)

(1): Sequential(

(0): BasicBlock(

level=None

(conv1): Conv2d(in_channels=64, out_channels=128, kernel_size=(3, 3), stride=(2, 2), padding=(1, 1), dilation=(1, 1), groups=1, level=None, bsgs_ratio=2.0)

(bn1): BatchNorm2d(level=None, level=None, fused=False)

(act1): ReLU(

level=None, degrees=[15, 15, 27]

(sign): _Sign(

level=None, degrees=[15, 15, 27]

(mult): Mult(level=None)

(acts): Sequential(

(0): Chebyshev(level=None, degree=15)

(1): Chebyshev(level=None, degree=15)

(2): Chebyshev(level=None, degree=27)

)

)

(mult1): Mult(level=None)

(mult2): Mult(level=None)

)

(conv2): Conv2d(in_channels=128, out_channels=128, kernel_size=(3, 3), stride=(1, 1), padding=(1, 1), dilation=(1, 1), groups=1, level=None, bsgs_ratio=2.0)

(bn2): BatchNorm2d(level=None, level=None, fused=False)

(act2): ReLU(

level=None, degrees=[15, 15, 27]

(sign): _Sign(

level=None, degrees=[15, 15, 27]

(mult): Mult(level=None)

(acts): Sequential(

(0): Chebyshev(level=None, degree=15)

(1): Chebyshev(level=None, degree=15)

(2): Chebyshev(level=None, degree=27)

)

)

(mult1): Mult(level=None)

(mult2): Mult(level=None)

)

(add): Add(level=None)

(shortcut): Sequential(

(0): Conv2d(in_channels=64, out_channels=128, kernel_size=(1, 1), stride=(2, 2), padding=(0, 0), dilation=(1, 1), groups=1, level=None, bsgs_ratio=2.0)

(1): BatchNorm2d(level=None, level=None, fused=False)

)

)

(1): BasicBlock(

level=None

(conv1): Conv2d(in_channels=128, out_channels=128, kernel_size=(3, 3), stride=(1, 1), padding=(1, 1), dilation=(1, 1), groups=1, level=None, bsgs_ratio=2.0)

(bn1): BatchNorm2d(level=None, level=None, fused=False)

(act1): ReLU(

level=None, degrees=[15, 15, 27]

(sign): _Sign(

level=None, degrees=[15, 15, 27]

(mult): Mult(level=None)

(acts): Sequential(

(0): Chebyshev(level=None, degree=15)

(1): Chebyshev(level=None, degree=15)

(2): Chebyshev(level=None, degree=27)

)

)

(mult1): Mult(level=None)

(mult2): Mult(level=None)

)

(conv2): Conv2d(in_channels=128, out_channels=128, kernel_size=(3, 3), stride=(1, 1), padding=(1, 1), dilation=(1, 1), groups=1, level=None, bsgs_ratio=2.0)

(bn2): BatchNorm2d(level=None, level=None, fused=False)

(act2): ReLU(

level=None, degrees=[15, 15, 27]

(sign): _Sign(

level=None, degrees=[15, 15, 27]

(mult): Mult(level=None)

(acts): Sequential(

(0): Chebyshev(level=None, degree=15)

(1): Chebyshev(level=None, degree=15)

(2): Chebyshev(level=None, degree=27)

)

)

(mult1): Mult(level=None)

(mult2): Mult(level=None)

)

(add): Add(level=None)

(shortcut): Sequential()

)

)

(2): Sequential(

(0): BasicBlock(

level=None

(conv1): Conv2d(in_channels=128, out_channels=256, kernel_size=(3, 3), stride=(2, 2), padding=(1, 1), dilation=(1, 1), groups=1, level=None, bsgs_ratio=2.0)

(bn1): BatchNorm2d(level=None, level=None, fused=False)

(act1): ReLU(

level=None, degrees=[15, 15, 27]

(sign): _Sign(

level=None, degrees=[15, 15, 27]

(mult): Mult(level=None)

(acts): Sequential(

(0): Chebyshev(level=None, degree=15)

(1): Chebyshev(level=None, degree=15)

(2): Chebyshev(level=None, degree=27)

)

)

(mult1): Mult(level=None)

(mult2): Mult(level=None)

)

(conv2): Conv2d(in_channels=256, out_channels=256, kernel_size=(3, 3), stride=(1, 1), padding=(1, 1), dilation=(1, 1), groups=1, level=None, bsgs_ratio=2.0)

(bn2): BatchNorm2d(level=None, level=None, fused=False)

(act2): ReLU(

level=None, degrees=[15, 15, 27]

(sign): _Sign(

level=None, degrees=[15, 15, 27]

(mult): Mult(level=None)

(acts): Sequential(

(0): Chebyshev(level=None, degree=15)

(1): Chebyshev(level=None, degree=15)

(2): Chebyshev(level=None, degree=27)

)

)

(mult1): Mult(level=None)

(mult2): Mult(level=None)

)

(add): Add(level=None)

(shortcut): Sequential(

(0): Conv2d(in_channels=128, out_channels=256, kernel_size=(1, 1), stride=(2, 2), padding=(0, 0), dilation=(1, 1), groups=1, level=None, bsgs_ratio=2.0)

(1): BatchNorm2d(level=None, level=None, fused=False)

)

)

(1): BasicBlock(

level=None

(conv1): Conv2d(in_channels=256, out_channels=256, kernel_size=(3, 3), stride=(1, 1), padding=(1, 1), dilation=(1, 1), groups=1, level=None, bsgs_ratio=2.0)

(bn1): BatchNorm2d(level=None, level=None, fused=False)

(act1): ReLU(

level=None, degrees=[15, 15, 27]

(sign): _Sign(

level=None, degrees=[15, 15, 27]

(mult): Mult(level=None)

(acts): Sequential(

(0): Chebyshev(level=None, degree=15)

(1): Chebyshev(level=None, degree=15)

(2): Chebyshev(level=None, degree=27)

)

)

(mult1): Mult(level=None)

(mult2): Mult(level=None)

)

(conv2): Conv2d(in_channels=256, out_channels=256, kernel_size=(3, 3), stride=(1, 1), padding=(1, 1), dilation=(1, 1), groups=1, level=None, bsgs_ratio=2.0)

(bn2): BatchNorm2d(level=None, level=None, fused=False)

(act2): ReLU(

level=None, degrees=[15, 15, 27]

(sign): _Sign(

level=None, degrees=[15, 15, 27]

(mult): Mult(level=None)

(acts): Sequential(

(0): Chebyshev(level=None, degree=15)

(1): Chebyshev(level=None, degree=15)

(2): Chebyshev(level=None, degree=27)

)

)

(mult1): Mult(level=None)

(mult2): Mult(level=None)

)

(add): Add(level=None)

(shortcut): Sequential()

)

)

(3): Sequential(

(0): BasicBlock(

level=None

(conv1): Conv2d(in_channels=256, out_channels=512, kernel_size=(3, 3), stride=(2, 2), padding=(1, 1), dilation=(1, 1), groups=1, level=None, bsgs_ratio=2.0)

(bn1): BatchNorm2d(level=None, level=None, fused=False)

(act1): ReLU(

level=None, degrees=[15, 15, 27]

(sign): _Sign(

level=None, degrees=[15, 15, 27]

(mult): Mult(level=None)

(acts): Sequential(

(0): Chebyshev(level=None, degree=15)

(1): Chebyshev(level=None, degree=15)

(2): Chebyshev(level=None, degree=27)

)

)

(mult1): Mult(level=None)

(mult2): Mult(level=None)

)

(conv2): Conv2d(in_channels=512, out_channels=512, kernel_size=(3, 3), stride=(1, 1), padding=(1, 1), dilation=(1, 1), groups=1, level=None, bsgs_ratio=2.0)

(bn2): BatchNorm2d(level=None, level=None, fused=False)

(act2): ReLU(

level=None, degrees=[15, 15, 27]

(sign): _Sign(

level=None, degrees=[15, 15, 27]

(mult): Mult(level=None)

(acts): Sequential(

(0): Chebyshev(level=None, degree=15)

(1): Chebyshev(level=None, degree=15)

(2): Chebyshev(level=None, degree=27)

)

)

(mult1): Mult(level=None)

(mult2): Mult(level=None)

)

(add): Add(level=None)

(shortcut): Sequential(

(0): Conv2d(in_channels=256, out_channels=512, kernel_size=(1, 1), stride=(2, 2), padding=(0, 0), dilation=(1, 1), groups=1, level=None, bsgs_ratio=2.0)

(1): BatchNorm2d(level=None, level=None, fused=False)

)

)

(1): BasicBlock(

level=None

(conv1): Conv2d(in_channels=512, out_channels=512, kernel_size=(3, 3), stride=(1, 1), padding=(1, 1), dilation=(1, 1), groups=1, level=None, bsgs_ratio=2.0)

(bn1): BatchNorm2d(level=None, level=None, fused=False)

(act1): ReLU(

level=None, degrees=[15, 15, 27]

(sign): _Sign(

level=None, degrees=[15, 15, 27]

(mult): Mult(level=None)

(acts): Sequential(

(0): Chebyshev(level=None, degree=15)

(1): Chebyshev(level=None, degree=15)

(2): Chebyshev(level=None, degree=27)

)

)

(mult1): Mult(level=None)

(mult2): Mult(level=None)

)

(conv2): Conv2d(in_channels=512, out_channels=512, kernel_size=(3, 3), stride=(1, 1), padding=(1, 1), dilation=(1, 1), groups=1, level=None, bsgs_ratio=2.0)

(bn2): BatchNorm2d(level=None, level=None, fused=False)

(act2): ReLU(

level=None, degrees=[15, 15, 27]

(sign): _Sign(

level=None, degrees=[15, 15, 27]

(mult): Mult(level=None)

(acts): Sequential(

(0): Chebyshev(level=None, degree=15)

(1): Chebyshev(level=None, degree=15)

(2): Chebyshev(level=None, degree=27)

)

)

(mult1): Mult(level=None)

(mult2): Mult(level=None)

)

(add): Add(level=None)

(shortcut): Sequential()

)

)

)

(avgpool): AdaptiveAvgPool2d(AdaptiveAvgPool2d(output_size=(1, 1)) level=None))

(flatten): Flatten(level=None, start_dim=1)

(linear): Linear(in_features=512, out_features=250, level=None, bsgs_ratio=2.0)

)

**Supplementary Figure 1.** | Impact of patch crop size on performance with the Target Expansion Strategy. Evaluation of $Approx\left( 224, 64 \right)_{250}$ and $Approx\left( 512, 64 \right)_{250}$ configurations. The plots compare Average PCC, SCC, and RMSE across top-ranked genes. $Approx\left( 224, 64 \right)_{250}$ consistently outperforms $Approx\left( 512, 64 \right)_{250}$ across all metrics. This confirms that the intermediate crop size of 224 provides the optimal field of view for the expanded prediction task.

**Supplementary Figure 2.** | Detailed PCC-based evaluation of privateST. (A) Gene-wise Pearson correlation coefficient (PCC) for privateST, EfficientNet_b0, and EfficientNet_b4. Results are shown for the average across all genes as well as 10 genes. (B) Distribution of PCC values across all genes for each model. The histogram illustrates the number of genes falling into each PCC range.

**Supplementary Figure 3.** | Qualitative visualization of input downsampling. Side-by-side comparison of an H&E-stained patch before and after the preprocessing step required for encryption efficiency (spot ID: BC23287_C1_10_17). (A) The left panel shows the original 224×224 pixel crop. (B) The right panel displays the same patch downsampled to 64×64 pixels via bilinear interpolation. Although extremely fine-grained nuclear textures are inevitably smoothed out by the resolution reduction, the primary morphological features—such as overall nuclear density, spatial cellular arrangement, and macroscopic tissue architecture—are preserved.

**Supplementary Figure 4**. | Overview for the mathematical space of privateST. First, the clinical researcher in a hospital encodes her message vector in $\mathbb{C}^{N/2}$ into a plaintext in a ring $R=\mathbb{Z[}X]/(X^{N}+1)$. The plaintext is encrypted into a ciphertext in $R_{q}^{2}$ using public key generated by the key management agency. Then, the client sends the ciphertext to the outsourced cloud server. The outsourced cloud server predicts the gene expression and sends the encrypted expression data to the hospital. Last, the clinical researcher decrypts the encrypted expression data using the secret key and decodes the result into a message vector in $\mathbb{C}^{N/2}$.

**Supplementary Table 1**

**Supplementary Table 1** provides a comprehensive, gene-by-gene breakdown of the performance metrics for the three models evaluated in the main text: privateST, EfficientNet_b0, and EfficientNet_b4. The table includes the Pearson Correlation Coefficient (PCC), Spearman Correlation Coefficient (SCC), and Root Mean Square Error (RMSE) for each of the 100 target genes. The performance metrics of all three models for all 100 genes are provided in **Supplementary Table 1**, which is attached as a separate Excel file.

**Reference**

1.	Yuan, X., and Rosen, J.M. (2025). Histone acetylation modulators in breast cancer. Breast Cancer Research *27*, 49. 10.1186/s13058-025-02006-9.

2.	Ayoubi, H.A., Mahjoubi, F., and Mirzaei, R. (2017). Investigation of the human H3.3B (H3F3B) gene expression as a novel marker in patients with colorectal cancer. J Gastrointest Oncol *8*, 64–69. 10.21037/jgo.2016.12.12.

3.	Kirkiz, E., Meers, O., Grebien, F., and Buschbeck, M. (2023). Histone Variants and Their Chaperones in Hematological Malignancies. Hemasphere *7*, e927. 10.1097/hs9.0000000000000927.

4.	Kleiner, I. (1996). The Genesis of the Abstract Ring Concept. Mathematical Association of America *103*, 417–424.

5.	CORRY, L. (2000). THE ORIGINS OF THE DEFINITION OF ABSTRACT RINGS. Modern Logic *8*, 5–27.
